# Knockdown of eIF3a alleviates pulmonary arterial hypertension by inhibiting endothelial-to-mesenchymal transition via TGFβ1/SMAD pathway

**DOI:** 10.1186/s12967-025-06505-3

**Published:** 2025-05-09

**Authors:** Qiuhong Jiao, Xiufeng Xu, Longwu Xu, Yuying Wang, Shulan Pang, Jie Hao, Xiaohong Liu, Yudan Zhao, Wanpeng Qi, Limin Qin, Tao Huang, Jingtian Li, Tao Wang

**Affiliations:** 1 Department of Cardiology, Affiliated Hospital of Shandong Second Medical University, Weifang, Shandong China; 2 Department of Geriatrics, Affiliated Hospital of Shandong Second Medical University, Weifang, Shandong China

**Keywords:** WGCNA, Pulmonary arterial hypertension, Pulmonary artery endothelial cells, TGFβ1/SMAD, Endothelial-to-mesenchymal transition

## Abstract

**Objective:**

Pulmonary arterial hypertension (PAH) is a life-threatening disease characterized by vascular remodeling and involves Endothelial-to-Mesenchymal transition (EndMT) in pulmonary artery endothelial cells (PAECs). EndMT is a complex cell differentiation process, mainly showing the detachment of endothelial cell migration and reducing endothelial cell characteristics to varying degrees, acquiring mesenchymal cell characteristics. In addition, numerous studies have reported that eIF3a over expression plays an important role in the occurrence and development of fibrotic diseases, cancer, and degenerative lesions, however, the mechanisms of eIF3a affecting the dysfunction of pulmonary arterial endothelial cells remains largely unknown. Therefore, we aimed to demonstrate the underlying mechanisms of eIF3a-knockdown inhibiting EndMT by regulating TGFβ1/SMAD signal pathway in PAH.

**Methods:**

In this study, we screened the potential target genes associated with idiopathic pulmonary arterial hypertension (IPAH) by WGCNA to provide a reference for the diagnosis and treatment of PAH. By constructing WGCNA, which indicated that the blue module (module-trait associations between modules and clinical feature information were calculated to selected the optimum module) is most closely associated with IPAH, we further screened out 10 up-regulated candidate biomarker genes. Male SD rats were randomly assigned to four groups: Control, Monocrotaline (MCT), AAV1-shRNA-NC group and AAV1-shRNA-eIF3a group. The eIF3a-knockdown rat model was constructed by adeno-associated virus type-1 (AAV1) infection, PAH was evaluated according to hemodynamic alteration, right heart hypertrophy and histopathological changes in the lung tissue. Hematoxylin eosin (H&E) staining was used to assess the morphological changes of pulmonary arteries in rats of each treatment group. Co-localization of eIF3a with alpha-small muscle action (α-SMA) and co-localization of eIF3a with endothelial marker (CD31) were detected by double-label immunofluorescence. Immunohistochemistry (IHC) and Western blot (WB) experiments were performed to assess the expression of eIF3a, EndMT and TGFβ1/SMAD signal related proteins. In vitro, primary rat pulmonary artery endothelial cells (PAECs) were transfected with si-eIF3a to investigate the effects of eIF3a-knockdown on hypoxia-induced EndMT in PAECs and further elucidate its underlying molecular mechanisms.

**Results:**

By WGCNA analysis, we screened the up-regulated hub genes of *TMF1, GOLGB1, ARMC8, PRPF40 A, EIF3 A, ROCK2, EIF5B, CCP110*, and *KRR1* associated with PAH, and in order to verify the potential role of eIF3a in the development of pulmonary arterial hypertension, MCT-induced PAH rat model was constructed successfully. The expression of eIF3a was increased in MCT-treated lungs. Knockdown of eIF3a significantly inhibited the pulmonary arterial hypertension and vascular remodeling in MCT-induced PAH rat model, ameliorated MCT-induced increases of right ventricular systolic pressure (RVSP) and right ventricular hypertrophy (RVH) in rats. Double-labeled immunofluorescence showed eIF3a was mostly co-localized with CD31, this result indicated that the development of MCT-induced PAH was related to the regulation of PAECs function (most likely associated with the change of EndMT in endothelial cells). WB showed that the expressions of EndMT related proteins were significantly increased by regulating TGFβ1/SMAD signaling pathway in MCT-induced PAH rat lung tissues, however, knockdown of eIF3a markedly attenuated these changes. In addition, we observed the same results in rat PAECs with chronic hypoxia exposure. These results indicate that eIF3a-knockdown inhibited EndMT by regulating TGFβ1/SMAD signaling pathway in PAECs, thereby improving the development of MCT-induced PAH.

**Conclusions:**

Knockdown of eIF3a inhibited EndMT in PAECs regulating TGFβ1/SMAD signaling pathway, significantly alleviated the changes of RVSP, RVH and vascular remodeling in MCT-induced PAH rats, eIF3a may be a promising and novel therapeutic target for the treatment of PAH.

**Supplementary Information:**

The online version contains supplementary material available at 10.1186/s12967-025-06505-3.

## Introduction

Pulmonary arterial hypertension (PAH) is a serious and progressive lung disease, defined as a resting mean pulmonary artery pressure of 20 mmHg or above, which can lead to pulmonary arterial endothelial dysfunction and smooth muscular hypertrophy, resulting in impaired pliability and hemodynamics of the pulmonary vascular system, and right ventricular dysfunction [1–3]. Affected patients are often disabled by symptoms of dyspnea, fatigue, syncope and chest pain, and they are at high risk of right ventricular failure and premature death [4]. Numerous studies have shown that the development of PAH may be related to the role of endothelin-1, prostacyclin-mediated, vascular calcium channels and nitric oxide driven pathways [5–7]. With continuous research, EndMT has also received extensive attention in the development of pulmonary hypertension [8], but the specific mechanisms are still unclear owing to the complexity and interactions of signaling molecules in regulatory pathways.

Eukaryotic initiation Factor3 (eIF3) is the largest and most complex translation initiation factor and has been implicated in numerous steps of translation initiation, termination and ribosomal recycling [9, 10]. eIF3a is the largest subunit of eIF3, which is a key player in all steps of translation initiation, and may regulate the translation of a subset of mRNAs, cell cycle progression and the cell proliferation [11–13]. Many studies have reported the role of eIF3a over-expression on fibrosis disease, proliferation and differentiation of fibroblasts, for example, Guo et al. proposed that miR-497 suppresses TGF-β1-induced epithelial-mesenchymal transition (EMT) in alveolar epithelial cells by directly binding to the 3'UTR of eIF3a mRNA, thereby inhibiting eIF3a expression. This mechanism concurrently attenuates the TGF-β1-stimulated hyperproliferation of pulmonary fibroblasts and excessive extracellular matrix (ECM) deposition, highlighting the critical role of eIF3a downregulation in pulmonary fibrosis pathogenesis [14]. Li et al. demonstrated elevated eIF3a expression in bleomycin-induced pulmonary fibrosis rat models. Their investigation further revealed that exogenous TGF-β1 intervention significantly increased both eIF3a levels and extracellular regulated kinase 1/2 (ERK1/2) phosphorylation in pulmonary fibroblasts. These findings suggest that eIF3a exerts pro-fibrotic effects through ERK1/2 pathway activation in bleomycin-induced pulmonary fibrosis, with TGF-β1-mediated eIF3a upregulation being ERK1/2-dependent [15]. Li et al. revealed the crucial role of eIF3a in hypoxia-induced right ventricular remodeling through the regulation of TGF-β1-driven cardiac fibroblast proliferation and differentiation. This regulatory mechanism operates via the eIF3a/p27 signaling axis [16]. Moreover, studies have revealed that eIF3e functions as a novel regulator of hypoxia-inducible 2α (HIF2α) activity, suggesting that targeted silencing of eIF3e may hold therapeutic potential for treating hypoxia-related ischemic disorders, such as cardiac and cerebral ischemia, cutaneous injuries, and various vascular obstructive diseases [17–19]. With the increasing understanding of the physiological and pathological functions of eIF3a, it has gradually become important in cardiac and pulmonary fibrosis. However, its specific role in pulmonary hypertension and the detailed mechanism by which it regulates pulmonary artery endothelial cell function has yet to be identified.

The TGF-β superfamily comprises a large series of cytokine growth factors that control many cellular functions, including proliferation, migration, differentiation, and extracellular matrix secretion and deposition [20–22]. Under pathological conditions, the overexpression of TGF-β causes EndMT, epithelial-mesenchymal transition (EMT), ECM deposition, and cancer-associated fibroblast (CAF) formation, which can lead to fibrotic disease, and cancer [23, 24]. Smad2 and Smad3 are the two major downstream regulators that promote TGF-β1-mediated tissue fibrosis [25]. EndMT is an intricate cellular differentiation process whereby endothelial cells detach and migrate away from the endothelium, and to varying extents decrease endothelial properties and acquire mesenchymal features [26, 27]. EndMT has been implicated in several cardiovascular diseases, where TGFβ, inflammation and altered shear stress drive EndMT in the vascular endothelium [28]. Numerous studies have shown that EndMT plays an important role in the development of pulmonary arterial hypertension [29, 30], but the underlying mechanism needs further investigation. On the basis of the above discussion, the present study aimed to address the potential role of eIF3a in the pathological progression of pulmonary hypertension and its possible regulatory mechanisms in vitro and in vivo.

## Materials and methods

### Data collection

Microarray data set of IPAH (GSE113439) was downloaded from Gene Expression Omnibus (GEO) database at the National Center for Biotechnology Information (NCBI) (http://www.ncbi.nlm.nih.gov/geo/). This microarray profiling of 26 fresh frozen lung samples were obtained from the recipients organs of 15 patients with PAH and 11 normal controls (normal lung tissue obtained from tissue flanking lung cancer resections). Patients with non-idiopathic pulmonary hypertension were eliminated from the subsequent analysis. Therefore, this microarray profiling was obtained from human fresh frozen lung samples of 6 idiopathic pulmonary hypertension patients and 11 controls. Data tables of GPL6244 were used to annotate the series matrix files of GSE113439 with official gene symbols (i.e., replace the probe name with the official gene symbol), and the gene expression matrices were obtained.

### Data preprocessing and differentially expressed genes (DEGs) screening

We used the linear fit method, bayesian analysis and t‐test algorithm in the R package limma (version 3.36.5, Bioinformatics Division, The Walter and Eliza Hall Institute of Medical Research, Parkville, Victoria, Australia.) to identify the differentially expressed genes (DEGs) between PAH and the normal controls. |log_2_ (fold-change)|> 1 and adjusted *P* < 0.05 were set as thresholds of DEG screening.

### Co-expression network construction and identification of significant modules

The “WGCNA” R package was employed to construct co-expression network for the DEGs [31], WGCNA is a kind of soft threshold method to construct no scale systems biology approach and gene co-expression networks [32]. In this study, we performed WGCNA using fresh-frozen lung tissue samples obtained from the GEO database, including specimens from 6 patients diagnosed with idiopathic pulmonary arterial hypertension (IPAH) and 11 matched control subjects. Soft threshold power was selected through network topology analysis. The optimal soft threshold was chosen to convert the correlation matrix into an adjacency matrix, and a topological overlap matrix (TOM) was created from the adjacency matrix. And then, average-linkage hierarchical clustering was used for hierarchical clustering analysis of genes. After determining the gene modules by dynamic shear, we calculated the eigenvector values (module gigengene, ME) of each module in turn, and chose a cut line for module dendrogram and merged some module. To determine whether the modules were associated with IPAH, we then analyzed the importance of genes by t test.

### GO and pathway enrichment analyses

We performed Gene Ontology (GO) term and KEGG pathway analyses using the “cluster Profiler” package in R software for genes in each module, *P* < 0.05 was considered to be significant enrichment.

### Identification of hub genes

Gene module contains gene significance (GS) and module members (MM) two important concepts [33]. GS is the absolute value describing the relationship between genes and traits, showing the intra-module connectivity (IC) of each gene in the network and the biological significance of the module, IC value is obtained after intra-modular connectivity calculation, MM reveals the correlation between module characteristic genes and gene expression profile, indicating the importance of genes; MM value is obtained through module eigengene analysis. The hub genes were obtained after calculating the three parameter values of GS, IC and MM.

### Animals and treatments

All animal experiments were carried out with the approval of the Institutional Animal Care and Use Committee of Shandong Second Medical University (protocol code 2021SDL388). Male Sprague–Dawley (SD) rats (200–250 g body weight) were purchased from the Institute of Medical Laboratory Animal Center (Shandong, China) and kept in a specific pathogen-free barrier system with free access to the standard diet and water. Monocrotaline (MCT) is an 11-membered macrocyclic pyrrolizidine alkaloid (PA) derived from the seeds of the Crotalaria spectabilis plant. Among preclinical models of PAH, MCT animal model offers the advantage of mimic several key aspects of human PAH, including vascular remodeling, proliferation of smooth muscle cells, endothelial dysfunction, upregulation of inflammatory cytokines, and right ventricle failure, requiring a single drug injection [34]. In the preliminary experiment, normal control rats (n = 10) and MCT-induced PAH model rats (n = 10) were used to detect the differential expression of eIF3a. In the following experiment, SD rats were randomly divided into 4 groups: (I) Control group (n = 10), (II) MCT group (n = 10), (III) AAV1-shRNA-NC group (n = 10) and (IV) AAV1-shRNA-eIF3a group (n = 10). To establish PAH rat model, AAV1-shRNA-NC group, MCT group and AAV1-shRNA-eIF3a group were given a single subcutaneous injection of MCT at 50 mg/kg [35], the control group received saline. According to the expression and distribution of eIF3a in the previous experiments, AAV1 with ICAM2 (Vigene Biosciences, USA) was selected as the PAEC-specifific promoter. For AAV1-shRNA-eIF3a group and AAV1-shRNA-NC group, respectively, infected with AAV1 expressing green fluorescent protein (AAV1-shRNA-NC) or AAV1 containing rat eIF3a-shRNA (AAV1-shRNA-eIF3a) via tracheal injection (1 × 10^11^ viral genomes/rat). Control group and MCT group received saline. All rats were hemodynamically tested 4 weeks after infection and then were injected with pent obarbital sodium for euthanasia.

### Generation of recombinant AAV1-shRNA-eIF3a

Adeno-associated virus-1 vector (AAV1, Vigene Biosciences, USA) carrying eIF3a shRNA 5′-TACGATACAAGGCTGTTAGAGAG-3′ (named AAV1-shRNA-eIF3a) and carrying scramble sequence (named AAV1-shRNA-NC) were constructed by Genelily Biotech (Shanghai, China).

### Analysis of hemodynamic parameter

The right heart catheter method was used to detect hemodynamic changes. Right ventricular systolic pressure (RVSP), conventionally used as an indicator of mean pulmonary arterial pressure, was measured by 3 F polyethylene catheter and Power Lab data acquisition equipment (AD Instruments, Australia). And then, animals were euthanized, lungs and hearts were collected.

### Measurement of RVHI

Right ventricular hypertrophy index (RVHI) was measured to reflect right ventricular remodeling. The hearts were separated to the right ventricle (RV) and left ventricle plus septum (LV + S), RV and LV + S were weighed respectively, RVHI = RV/(LV + S). The ratio of RV weight to body weight (BW) was calculated as RV/BW.

### Pulmonary Arterial Morphometry

The lung tissues were separated from the experimental rats and fixed in 4% paraformaldehyde, embedded in paraffin, and then, the tissues were cut into 4–5 µm sections, the sections were subjected to H&E staining following standard procedures to assess the morphological changes of pulmonary arteries, the morphological damage of the tissues was observed by using a microscope. The percent medial wall thickness (WT%) was used to assess the extent of pulmonary vascular remodeling. WT% = (external diameter−internal diameter)/(external diameter) × 100%. All data were obtained and analyzed in a blinded manner.

### Immunohistochemistry (IHC)

Lung tissues were washed with PBS solution, and fixed by 4% paraformaldehyde for 24 h. Then, specimens were embedded in paraffin and cut into sections 4–5 μm thick, deparaffinized in xylene and rehydrated. To inhibit the activity of endogenous peroxidase, specimens were treated with 3% hydrogen peroxide for 10 min, and blocking with 5% goat serum albumin. The sections were incubated overnight at 4 °C with anti-eIF3a, and then incubated with goat anti-rabbit secondary antibody conjugated to peroxidase for 60 min at room temperature, followed by detection with the peroxidase substrate 3,3′-diaminobenzidine. The coloration process was monitored under a microscope.

### Immunofluorescence (IF)

For double immunofluorescence staining, paraffin embedded tissue sections were deparaffinized in xylene and rehydrated. Non-specifific binding sites of tissues sections were blocked with 5% bovine serum albumin (BSA) for 1 h at room temperature, and then, tissues incubated with mixed primary antibodies (eIF3a and CD31, 1:100 dilutions) and (eIF3a and α-SMA, 1:100 dilutions) at 4 °C overnight respectively. Primary antibodies were detected using goat anti-mouse Alexa Fluor-conjugated IgG (1:1000) and goat anti-rabbit FITC-conjugated IgG (1:1000) to stain for 1 h at room temperature, nuclei were stained with DAPI for 5 min. Images were obtained by fluorescence microscope (Olympus, Japan).

### PAECs culture and transfection

All primary rat PAECs were obtained from Science Cell (Shanghai, China) and maintained in endothelial cell medium (Shanghai, China) supplemented with 10% fetal bovine serum (Shanghai, China), 1% penicillin/streptomycin (Shanghai, China), and 1% endothelial cell growth supplement (Shanghai, China). Cells were cultured at 37 °C in a 5% CO_2_ incubator. Cells between passages 2 and 5 were used for experiments.

eIF3a-specific siRNA oligonucleotides were designed and purchased from Genelily Biotech (Shanghai, China). According to the instructions of lipofectamine 3000 transfection reagent, primary rat PAECs were transiently transfected with si-eIF3a or si-NC, cells were placed in 20% normoxia content and 2% hypoxia content 6 h later, and then cultured for another 24 h and used in subsequent experiments.

Rat PAECs were randomly divided into 4 experimental groups: (I) Control (Con), (II) eIF3a knockdown (si-eIF3a), (III) TGFβ inhibition (LY) and (IV) Combinatorial treatment (LY + si-eIF3a). Prior to hypoxia exposure, PAECs in the si-eIF3a and LY + si-eIF3a groups underwent siRNAsi-eIF3a transfection for 6 h. All PAECs were then cultured under 2% oxygen tension, with the LY and LY + si-eIF3a groups receiving additional treatment with 10 μM LY364947. Cells were harvested after 24 h for subsequent analyses [36, 37].

### Western blot

Immunoblotting was performed as previously described [38]. In brief, protein lysates were extracted from frozen lung samples or PAECs. A BCA Protein Assay Kit was used to determine the protein concentrations of the samples. Equal amounts of protein were separated by 8–16% SDS‒PAGE and then transferred onto PVDF membranes. The PVDF membranes were then blocked with 5% bovine serum albumin for 2 h at room temperature and incubated at 4 °C overnight with primary antibodies. After incubation with horseradish peroxidase-conjugated secondary antibodies for 60 min, immune reactive protein bands were detected by a BioSpectrum Gel Imaging System (UVP, California, USA) and quantified by Image J software. β-actin was used as a loading control for whole cellular protein.

### Antibodies and reagents

anti-eIF3a (#ab128996, Abcam, Cambridge, MA); anti-CD31 (#ab64543, Abcam, Cambridge, MA); anti-α-SMA (#ab7817, Abcam, Cambridge, MA); anti-CollagenI (#ab316222, Abcam, Cambridge, MA); anti-CollagenIII (#ab184993, Abcam, Cambridge, MA); anti-Vimentin (#ab92547, Abcam, Cambridge, MA); anti-TGFβ1 (#ab315254, Abcam, Cambridge, MA); anti-SMAD2/3 (#ab202445, Abcam, Cambridge, MA); anti-P-SMAD2/3 (#AF3367, Affinity Biosciences, China); anti-Snail (#ab180714, Abcam, Cambridge, MA); anti-Slug (#ab27568, Abcam, Cambridge, MA); anti-Twist1 (#ab175430, Abcam, Cambridge, MA); anti-β-actin (#ab179467, Abcam, Cambridge, MA); goat anti-mouse Alexa Fluor-conjugated IgG (#a0473, Beyotime, China); goat anti-rabbit FITC-conjugated IgG (#a0568, Beyotime, China); MCT (# C2401, Sigma‒Aldrich, St. Louis, MO, USA); lipofectamine 3000 (L3000015, Thermo Fisher Scientific, USA); BCA Protein Assay Kit (#a65453, Thermo Fisher Scientific, USA); LY2109761 (LY2109761, MedChemExpress, USA); PVDF (HVLP09050, Merck Millipore, MA, USA).

### Statistical analysis

Graph Pad Prism 8.0 (GraphPad, USA) statistical software was used for data analysis, and one-way analysis of variance (one-way ANOVA) followed by Tukey’s multiple comparison test was used for comparisons among multiple groups. A t-test was used to analyze differences between two groups. Most bioinformatics analyses including WGCNA were performed in R with default test statistics and cutoff values as specified in individual method sections. Data are expressed as the mean ± standard deviation (x ± s), and statistical significance was accepted at values of *P* < 0.05.

## Results

### DEGs selection and functional enrichment analysis of DEGs

**Fig. 1:**
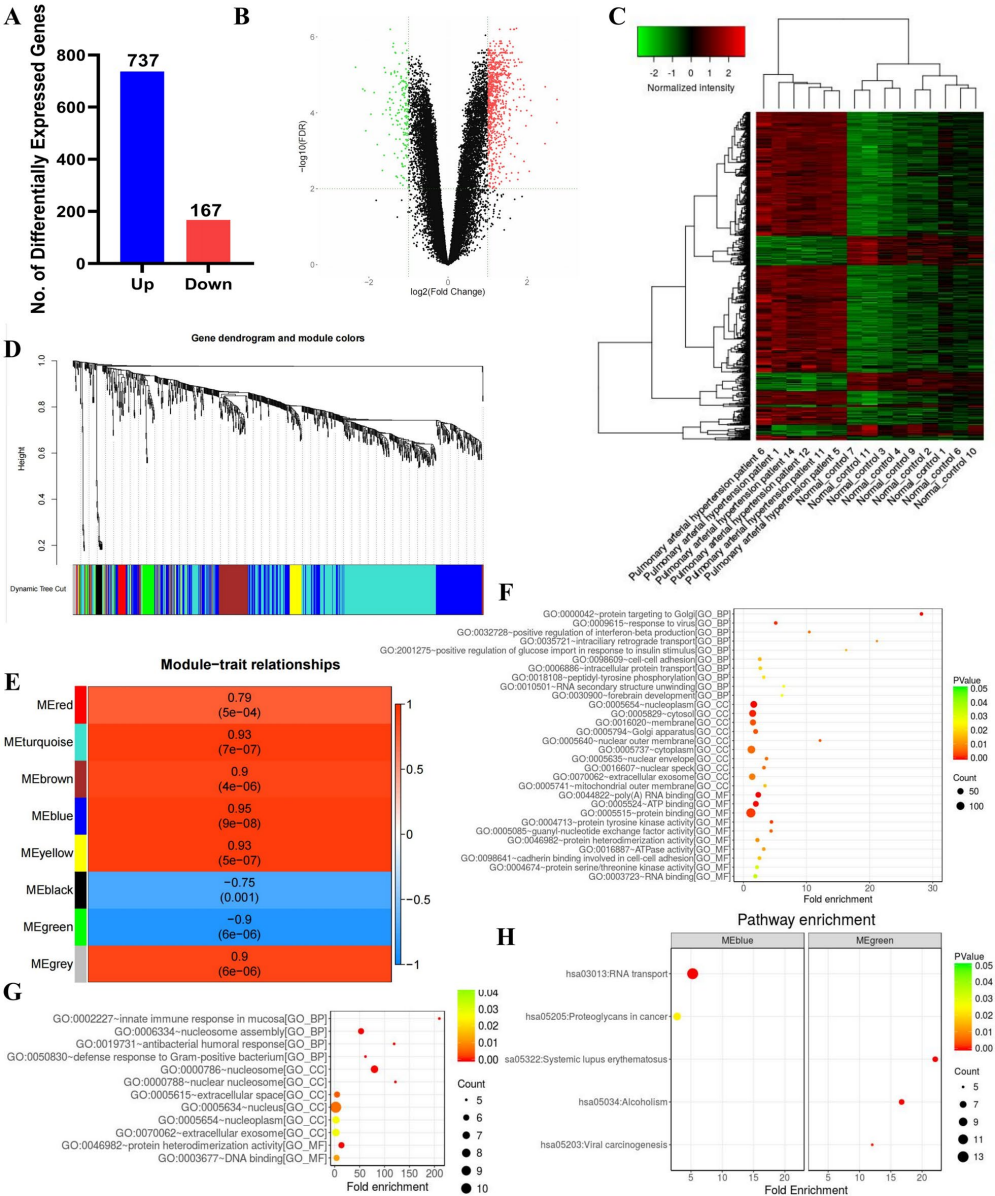
Differential expressed genes analysis. **A** Statistical histograms of the DEGs, blue bar represent the number of upregulated genes and red bar represent the number of downregulated genes. **B** Volcano plot of DEGs, set |log_2_(fold-change)|> 1 and *P* < 0.05 as significance criteria. Red dots are upregulated genes, and green dots are downregulated genes. **C** Heatmap of DEGs, the vertical axis represents DEGs, the horizontal axis represents sample. Red represents upregulated genes and green represents downregulated genes. **D** Clustering dendrogram of the genes involved. **E** Heatmap of the correlations between MEs and clinical traits. Correlation coefficients and corresponding P-values are shown in the rectangles and the brackets, respectively. **F** Dot plot of GO analysis for blue module DEGs. **G** Dot plot of GO analysis for green module DEGs. **H** Dot plot of KEGG pathway enrichment analysis for blue and green module DEGs

We replaced the gene data processed by gene annotation and data with the limma package in R. A total of 904 DEGs between the IPAH group and the control group were identified with thresholds of |log_2_(fold-change)|> 1 and *P* < 0.05, and consisted of 167 downregulated genes and 737 upregulated genes (Fig. 1A). We then constructed a volcano plot of the DEGs (Fig. 1B) and the gene expression heatmap (Fig. 1C), which revealed that among the 904 DEGs between the IPAH and control groups, the general gene expression patterns clearly differed between the two groups. We used the Soft Threshold function in WGCNA, which performs network topology analysis. After the calculation, we selected β = 29 to construct gene modules using the WGCNA package since the scale independence reached 0.8 and had relatively high average connectivity (Fig. S4 A-D). After determining the soft threshold, a total of 737 DEGs were used to construct weighted gene co-expression networks. Finally distinguishing the set of 8 co-expression modules, expressed as red, turquoise, brown, blue, yellow, black, green and grey (Fig. 1D). To identify the clinically significant modules associated with IPAH, the Pearson’s correlations between the MEs of the 8 modules and the clinical traits were obtained (Fig. 1E). Moreover, GS for IPAH was also calculated in each module. The mean absolute values of GS in each module were calculated and visualized, suggesting that the blue module was the clinically significant module with the highest GS for IPAH (Fig. S4E). According the modul–trait relationships, the ME of the green module (r = − 0.90; *P* = 6E − 06) showed the higher corrections with IPAH and was negatively correlated with the disease, while ME blue was positively correlated with the disease and the ME of the blue module (r = 0.95; *P* = 9E − 08) showed the highest corrections with IPAH. Furthermore, the MM values were obtained, followed by the correlation analysis between the GS for IPAH and the MM for genes in the 8 modules, respectively, GS and MM of each gene are shown in Figure S4 F-G. GO enrichment analysis of DEGs was performed. The results of these analyses showed that, for the blue module, the GO enrichment results of DEGs were presented in Fig. 1F. For the green module, the GO enrichment results of DEGs were presented in Fig. 1G. In order to understand the enrichment of the pathway, KEGG analysis was used to analyze the pathway, and it was found that these molecules of blue module were mainly involved in RNA transport and these molecules of green module were mainly involved in Systemic lupus erythematosus (Fig. 1H).

In this study, 10 genes (*CUL3, TMF1, GOLGB1, ARMC8, PRPF40 A, EIF3 A, ROCK2, EIF5B, CCP110* and *KRR1*) with high connectivity in the dodger blue module and 5 genes (*TMEM258, NDUFA3, HINT2, PRDX5* and *SNHG8/SNORA24*) with high connectivity in the dodger green module were considered as hub genes (Fig. S1-2). As illustrated in Figure S1, blue module analysis revealed that eIF3a not only has significant differential expression patterns but also has established associations with fibrotic disease pathogenesis in previous studies. Building on these findings, our current investigation focused on elucidating the functional relationship between eIF3a and pulmonary arterial hypertension progression, with a particular emphasis on uncovering its underlying molecular mechanisms.

### Establishment of PAH Rat Model Induced by MCT

**Fig. 2:**
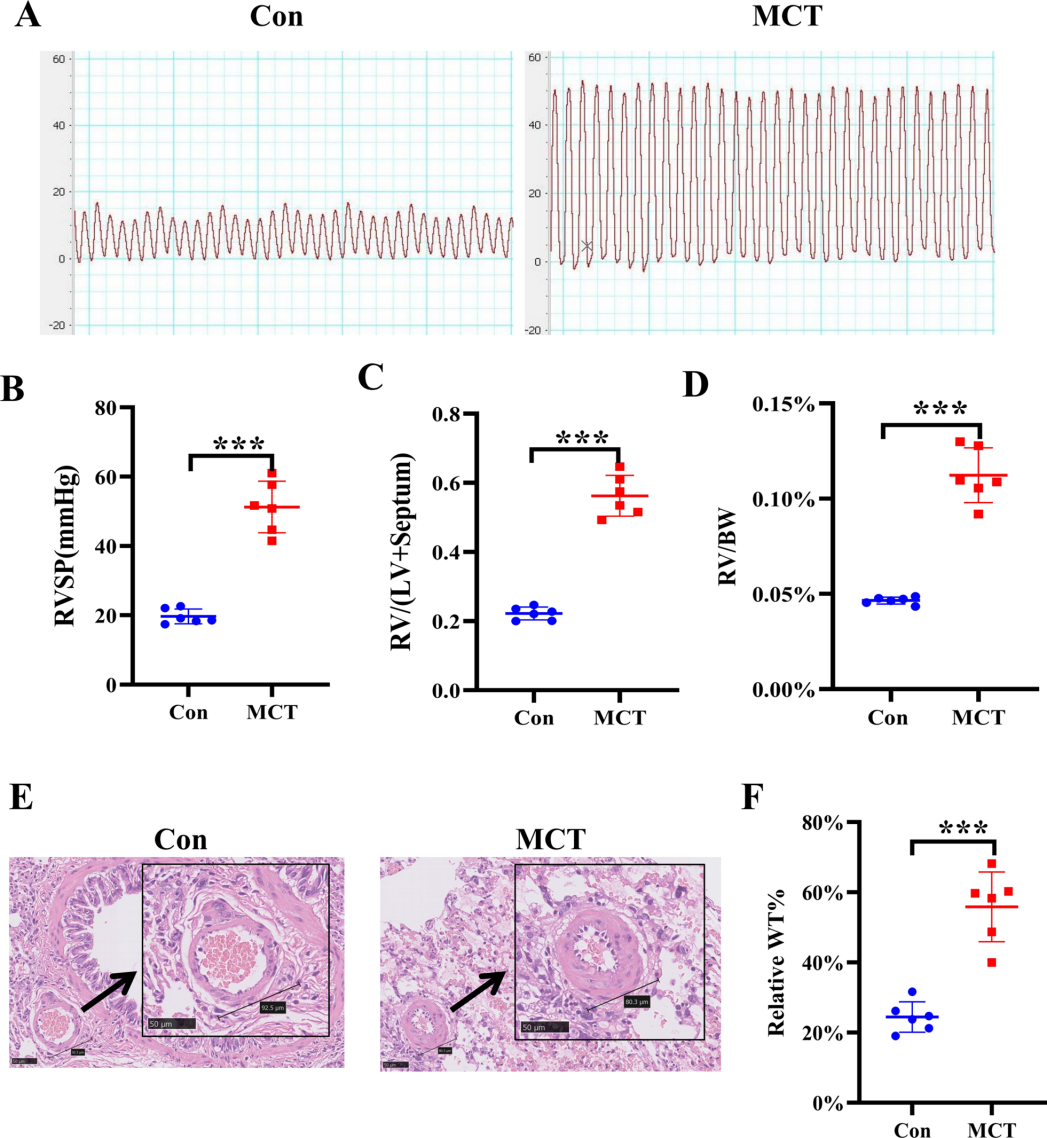
Establishment of a PAH rat model induced by MCT. **A** Representative image of RVSP measurement. **B** Summarized data of RVSP. **C** Summarized data of right ventricle/left ventricle plus septum. **D** Summarized data of right ventricle/body weight. **E** Summarized data of WT%. **F** Representative image of H&E staining (Scale bar: 50 μm). Data are represented as the mean ± SD, n = 6–10, ***P < 0.001 vs. Control group

Pulmonary hypertension was evaluated in terms of right ventricular systolic pressure (RVSP), as shown in Fig. 2A, B, MCT resulted in a significant increase in RVSP compared with that in the control group after 4 weeks of MCT administration (51.26 ± 7.428 mmHg vs. 19.71 ± 2.130 mmHg, *P* < 0.001). Similarly, the right ventricle/left ventricle plus septum (RV/LV + S) (0.5626 ± 0.05905 vs. 0.2221 ± 0.01869, *P* < 0.001) and RV/BW (0.1123 ± 0.01437 vs. 0.04650 ± 0.0001869, *P* < 0.001) ratios were significantly greater in the MCT group than in the control group (Fig. 2C, D). To determine the effect of vascular remodeling in the MCT-induced PH model, we conducted histological studies. Distal pulmonary arterioles with a diameter of 50–200 μm were analyzed in lung slices stained with H&E (Fig. 2E). MCT administration resulted in significant vascular remodeling with remarkable medial wall thickening, which is a key characteristic of PAH compared with that in the control group (Fig. 2F). The results showed that the PAH model was well established.

### eIF3a overexpression in the MCT-induced PAH rat model

**Fig. 3:**
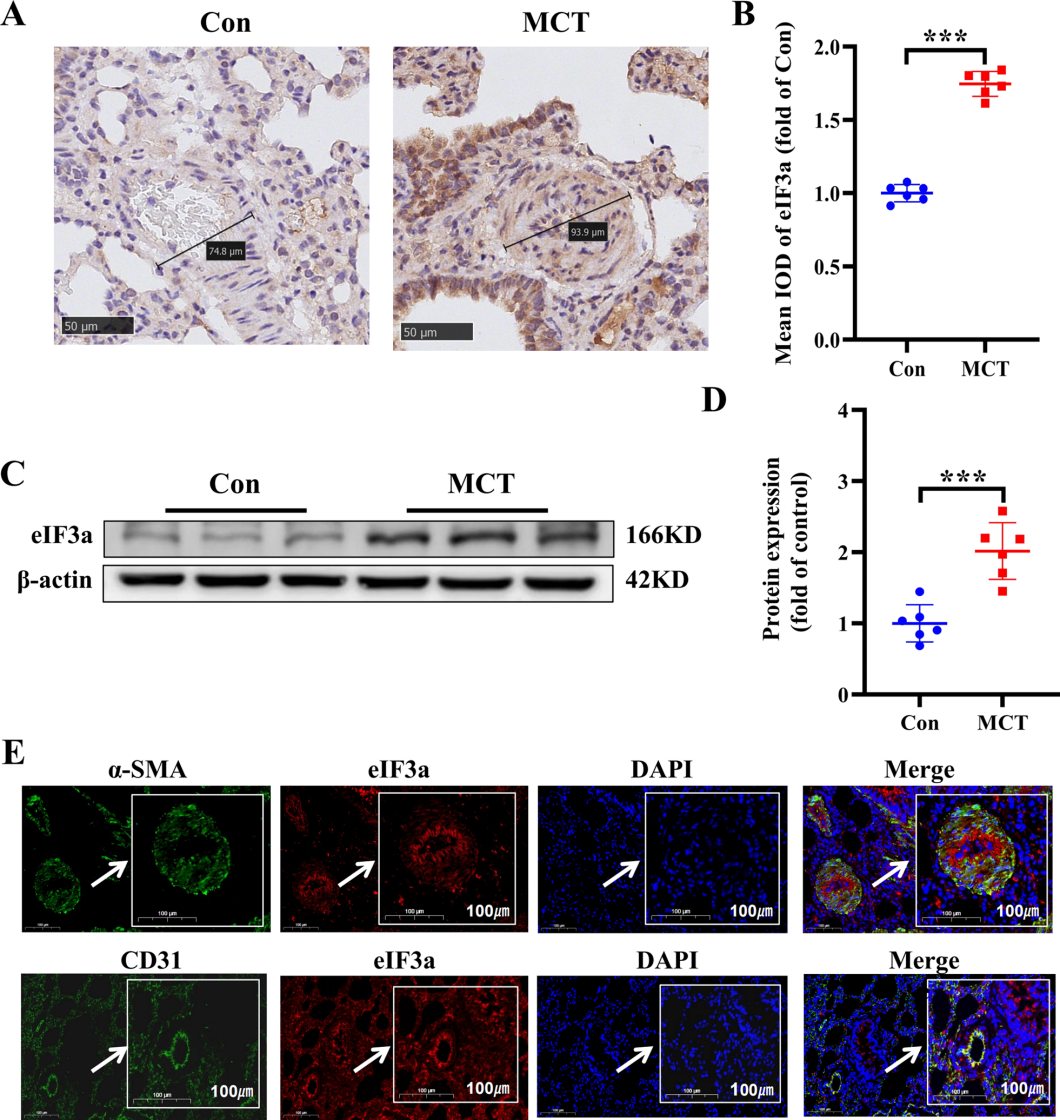
Expression and localization of eIF3a in MCT-induced PAH rat model. **A** Representative image of immunohistochemical staining of eIF3a (Scale bar: 50 μm). **B** Summarized data of immunohistochemical staining of eIF3a. **C**, **D** Western blot and the statistical result of eIF3a proteins in lung tissues. **E** Immunofluorescence examination indicated the location profile of eIF3a in pulmonary arteries (Scale bar: 100 μm). Data are represented as the mean ± SD, n = 6–10, ***P < 0.001 vs. Control group

To confirm that eIF3a is involved in the remodeling of the pulmonary arterial wall, we tested the expression of eIF3a in an MCT-induced PAH rat model. Immunohistochemical staining of eIF3a revealed that, compared with that in the control group, the expression of eIF3a in the MCT group was notably greater (Fig. 3A, B). To further verify the role of eIF3a in the development of PAH, WB experiments were performed, and the results indicated that the protein level of eIF3a increased nearly twofold in the MCT group compared with that in the control group (Fig. 3C, D). Microarray data sets of IPAH (GSE15197, GSE53408) were downloaded from Gene Expression Omnibus (GEO) database, Similarly, microarray datasets related to IPAH were obtained from the GEO database (accession numbers GSE15197 and GSE53408). Subsequent analysis revealed significant upregulation of eIF3a expression in pulmonary hypertension tissues compared to normal controls (Fig. S4 A-B). Furthermore, we detected the location of eIF3a in the small pulmonary artery. Immunofluorescence examination revealed that eIF3a was significantly colocalized with CD31 (pulmonary artery endothelial cell-labeled) but was absent or weakly colocalized with α-SMA (pulmonary artery fibroblast-labeled) (Fig. 3E). In summary, MCT induced pathological remodeling of the pulmonary artery and right ventricular hypertrophy in the PAH rat model, and these alterations may be related to the high expression of eIF3a in pulmonary arterial endothelial cells.

### Knockdown of eIF3a prevented changes in pulmonary hemodynamics and pathological remodeling of the pulmonary artery in PAH rats

**Fig. 4:**
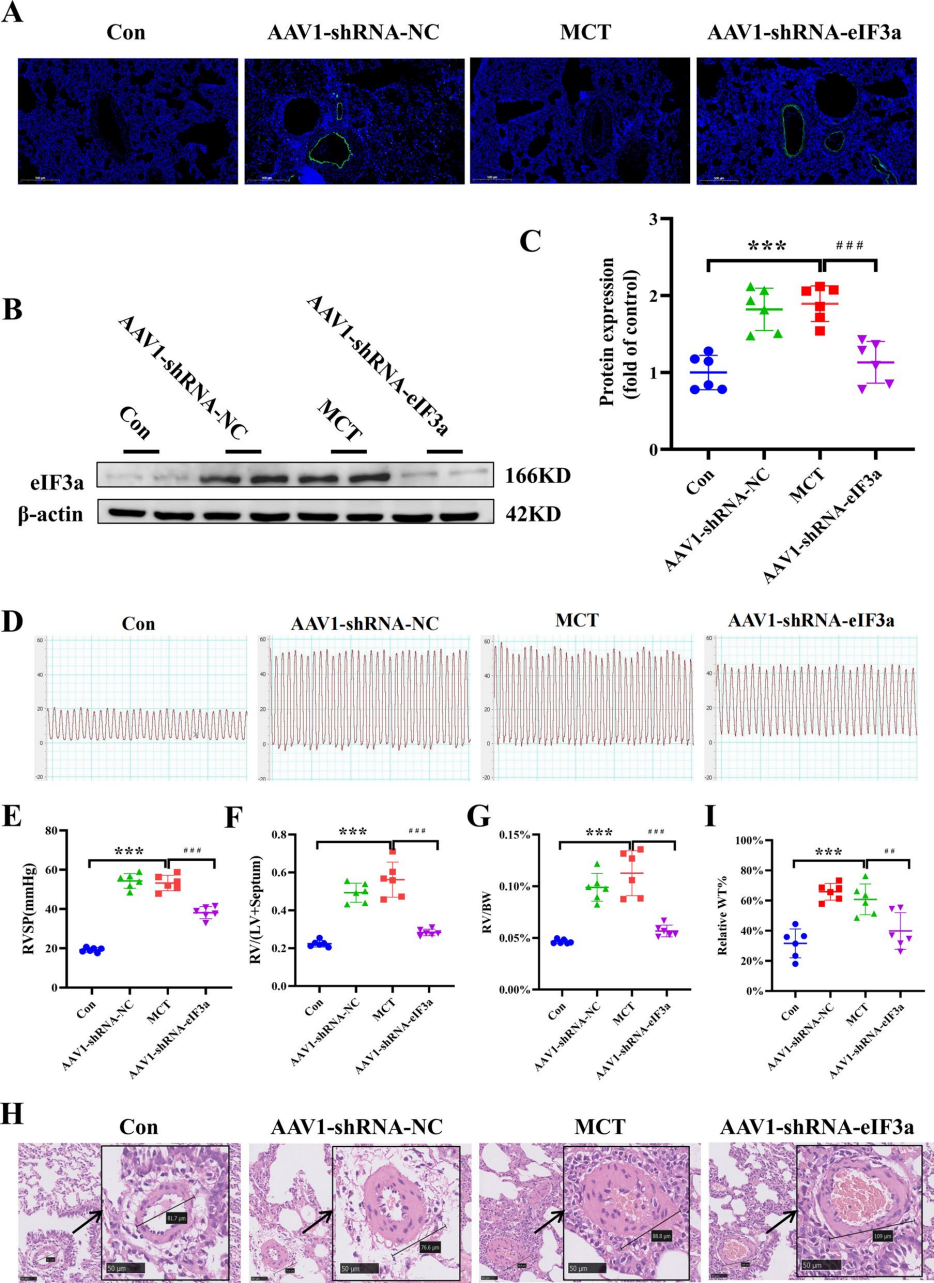
Knockdown of eIF3a ameliorated pulmonary hypertension induced by MCT. **A** Image captured by fluorescence microscopy to trace the location and expression of injected AAV in rodent lung tissues. **B**, **C** Western blot and the statistical result of eIF3a proteins. **D** Representative image of RVSP measurement. **E** Summarized data of RVSP. **F** Summarized data of right ventricle/left ventricle plus septum. **G** Summarized data of right ventricle/body weight. **H** Representative image of H&E staining (Scale bar: 50 μm). **I** Summarized data of WT%. Data are represented as the mean ± SD, n = 6–10, ***P < 0.001 vs. Control group, ^###^*P* < 0.001 vs. MCT group

To explore the role of eIF3a in the MCT-induced PAH rat model, the rats were subjected to intratracheal injection of AAV1-carried scrambled shRNA (AAV1-shRNA-NC) as a negative control or AAV1-carried eIF3a shRNA (AAV-shRNA-eIF3a) to knock down the expression of eIF3a. Since AAV1 was designed to express enhanced green fluorescence protein (eGFP) as a reporter gene, we were able to trace the location of the injected virus and confirm its expression in pulmonary vessels (Fig. 4A). To test the expression of eIF3 in the lung tissues of PAH rats treated with AAV1-shRNA-NC and AAV1-shRNA-eIF3a, we performed WB experiments, and the results are shown in Fig. 3B. The expression of eIF3a was significantly greater in the pulmonary tissues of PAH rats treated with AAV 1-shRNA-NC than in those of the control group, whereas eIF3a was significantly lower in AAV-shRNA-eIF3a-treated PAH rats than in those of the MCT group. Hemodynamic tests revealed that right ventricular systolic pressure was significantly increased in the MCT group and the AAV1-shRNA-NC group, but only a slight increase was detected in the AAV-shRNA-eIF3a group (Fig. 4B, C). In addition, the RV/(LV + S) ratio and RV/BW ratio were significantly greater in the MCT group than in the AAV1-shRNA-NC group. After treatment with AAV-shRNA-eIF3a, the RV/(LV + S) ratio and RV/BW ratio were comparable to those in the control group (Fig. 4D–G). The hallmark of PAH is pulmonary vascular remodeling, which is characterized by increased pulmonary artery medial thickness. To observe morphological changes in pulmonary arterioles, we performed H&E staining experiments, and the results indicated that the lumens of pulmonary arterioles in the MCT group and AAV1-shRNA-NC group were nearly obstructed, whereas the vascular medial wall became mildly thick in the AAV-shRNA-eIF3a group (Fig. 4H, I). In short, the knockdown of eIF3a improved hemodynamic deterioration in an MCT-induced PH model, alleviating the elevated RVSP and pathological remodeling of the pulmonary artery.

### Knockdown of eIF3a prevented the change in EndMT and deposition of the extracellular matrix in PAH rats

**Fig. 5:**
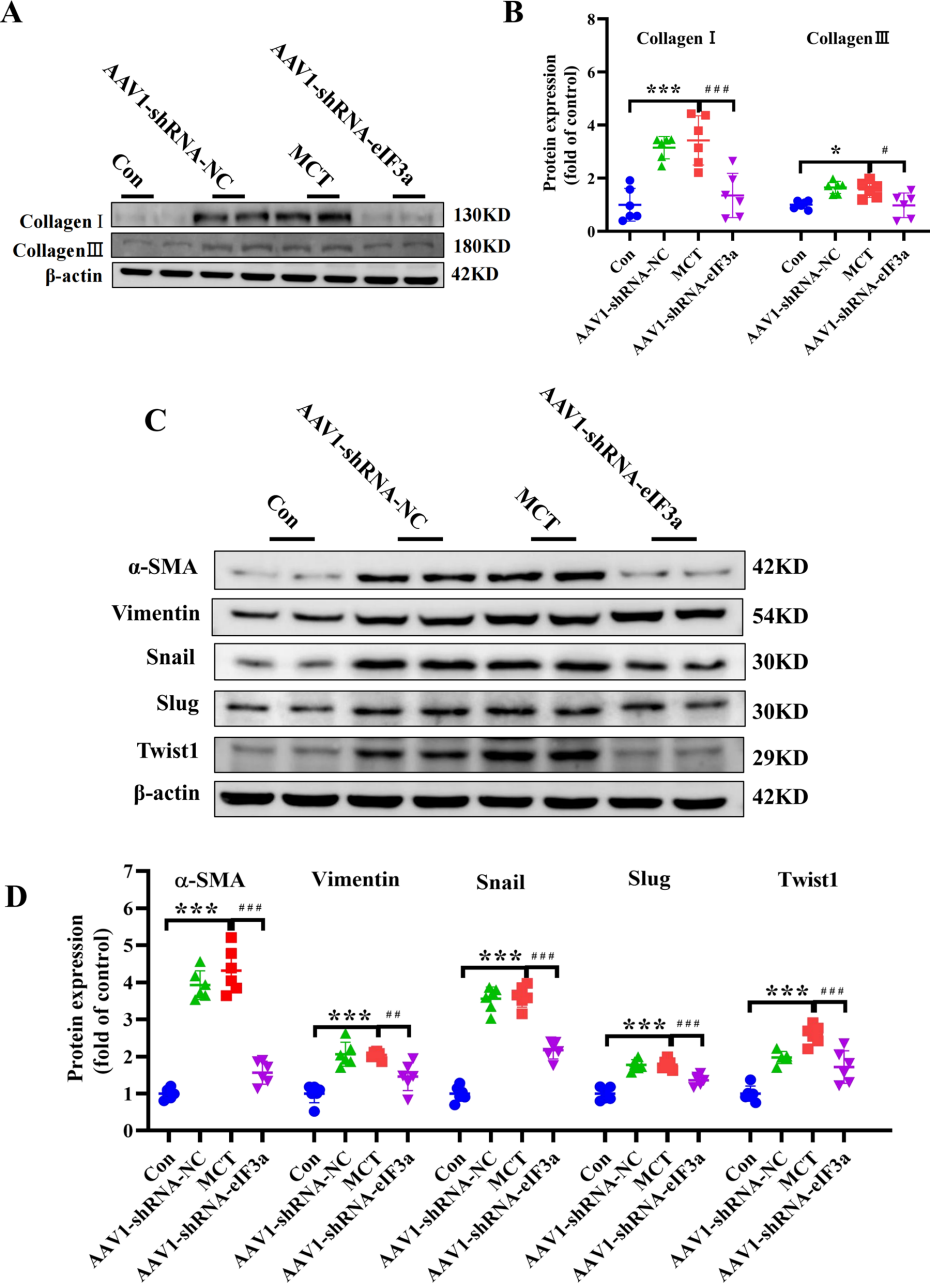
Knockdown of eIF3a prevented EndMT and deposition of the extracellular matrix in PAH rats. **A**, **B** Western blot and the statistical results of Collagen I and Collagen III proteins. **C**, **D** Western blot and the statistical result of α-SMA, Vimentin, Snail, Slug and Twist1 proteins. Data are represented as the mean ± SD, n = 6–10, *P < 0.05, ***P < 0.001 vs. Control group, ^#^*P* < 0.05, ^# #^*P* < 0.01, ^# # #^*P* < 0.001 vs. MCT group

Myofibroblasts in the pulmonary artery may result from the phenotypic conversion of endothelial cells into activated myofibroblasts, a process known as endothelial-to-mesenchymal transition (EndMT). Recently, it has been postulated that EndMT may play a role in the development of PAH, we performed WB experiments to explore the role of EndMT in the development of PAH, as shown in Fig. 5A, B, Collagen I and collagen III, the main components of the extracellular matrix (ECM), were notably increased in the MCT group and MCT + AAV1-NC group, whereas these alterations were significantly inhibited by the knockdown of eIF3a. Myofibroblasts are considered to responsible for the production of ECM during fibrosis, as specific markers of myofibroblasts, our experimental results demonstrated that both α-SMA and vimentin protein expression levels were markedly elevated in the MCT-induced pulmonary hypertension group. Notably, eIF3a knockdown significantly attenuated the pulmonary hypertension-induced upregulation of these myofibroblast markers. Furthermore, EndMT activation has been associated with myofibroblast production. In the present study, the knockdown of eIF3a was found to downregulate α-SMA and vimentin protein levels. Snail, slug and twist1 are key regulators of the EndMT program, whereas the knockdown of eIF3a attenuated the MCT-induced upregulation of snail, slug and twist1 in PAH rats (Fig. 5C, D). Our results showed that the knockdown of eIF3a can ameliorate the deposition of extracellular matrix components (including collagen III and collagen I). Furthermore, knockdown of eIF3a significantly suppressed EndMT, as evidenced by decreased expression of α-SMA, vimentin, Twist1, snail and slug.

### Hypoxia induces the upregulation of eIF3a in rat PAECs

**Fig. 6:**
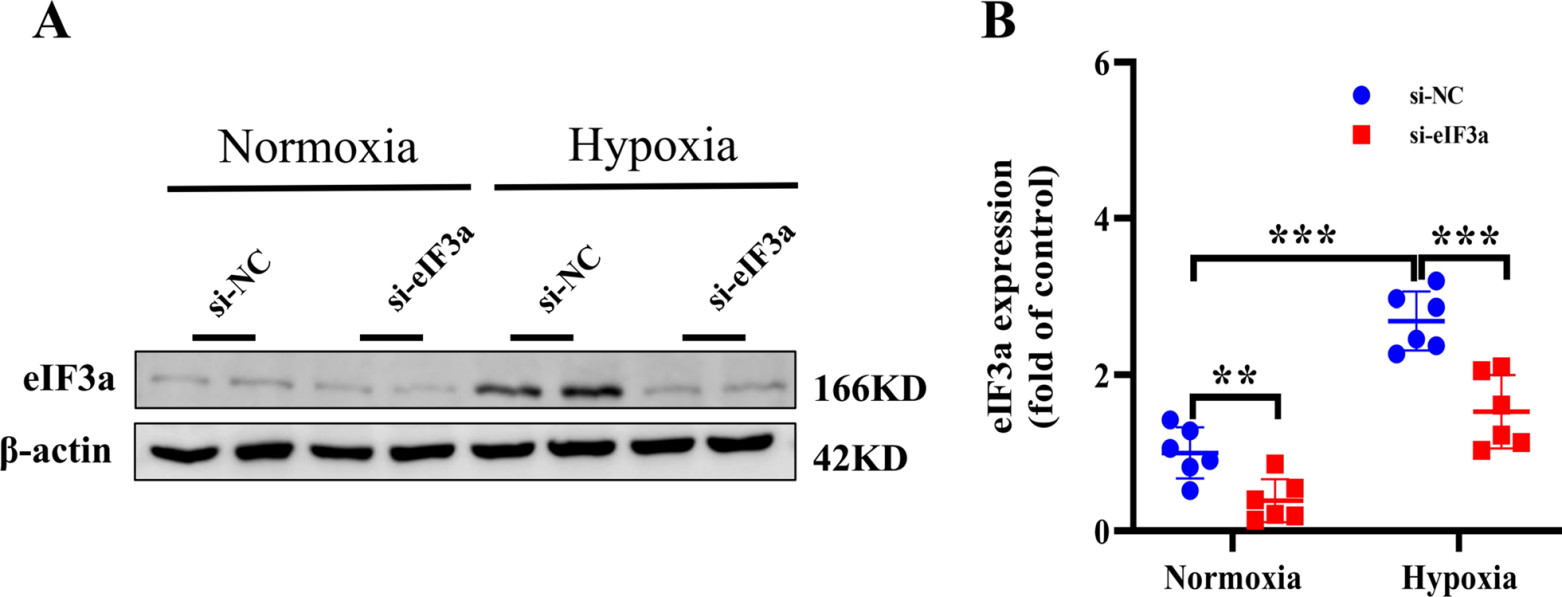
Hypoxia induced the upregulation of eIF3a in rat PAECs. **A**, **B** Western blot and the statistical results of eIF3a proteins. Data are represented as the mean ± SD, **P < 0.01, ***P < 0.001

To suppress eIF3a expression, rat pulmonary artery endothelial cells were treated with eIF3a-specific small interfering RNA (si-eIF3a) or si-NC, and 6 h after intervention, the cells were randomly divided into normoxic and hypoxic groups and then incubated under 20% normoxia and 2% hypoxia for 24 h. Then, the cells were harvested for protein isolation. To determine eIF3a expression under hypoxic conditions, the protein level was measured by western blotting. The results (Fig. 6A, B) revealed that, compared with normoxia, si-NC significantly increased the protein expression level of eIF3a in rat pulmonary arterial endothelial cells cultured in a hypoxic environment. Furthermore, in rat pulmonary artery endothelial cells cultured in a hypoxic environment, we observed that intervention with si-eIF3a significantly reversed the hypoxia-induced increase in eIF3a protein expression. Notably, in rat pulmonary arterial endothelial cells cultured under normoxia, compared with si-NC intervention, intervention with si-eIF3a significantly reduced eIF3a protein expression. These results indicate that hypoxia can induce the upregulation of eIF3a in rat pulmonary artery endothelial cells and that intervention with eIF3a-specific small interfering RNA can successfully knockdown eIF3a expression in these cells.

### Knocking down eIF3a alleviated the change in EndMT and deposition of the extracellular matrix induced by hypoxia in rat PAECs

**Fig. 7:**
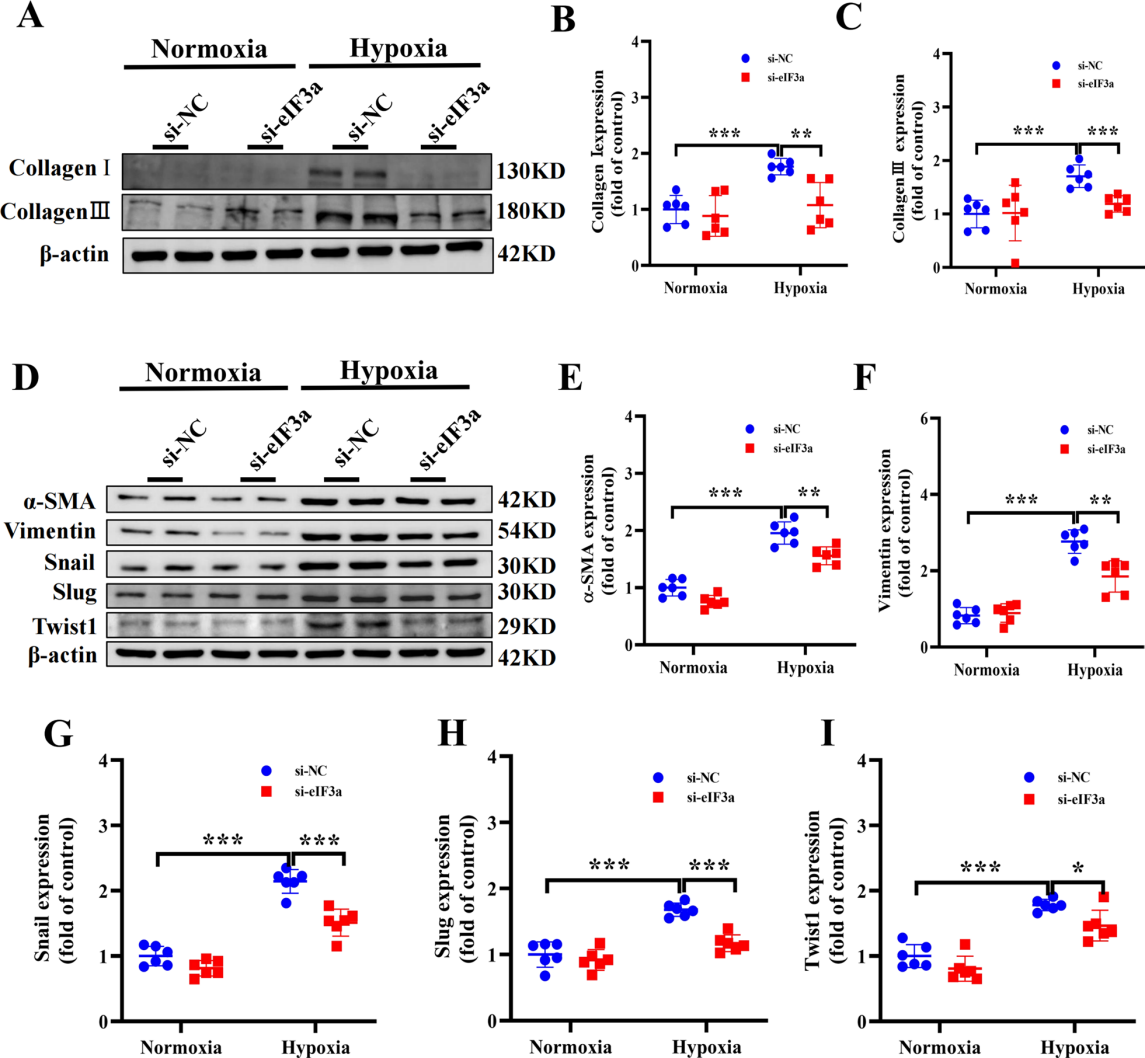
Knocking down eIF3a alleviated the change in EndMT and deposition of the extracellular matrix Induced by hypoxia in rat PAECs. **A**–**C** Western blot and the statistical results of proteins associated with the extracellular matrix deposition (Collagen I and Collagen III). **D**–**I** Western blot and the statistical results of proteins associated with EndMT (α-SMA, Vimentin, Snail, Slug and Twist1). Data are represented as the mean ± SD, *P < 0.05, **P < 0.01, ***P < 0.001

To further explore the effects of eIF3a on the changes in EndMT and deposition of the extracellular matrix induced by hypoxia in rat PAECs, several relevant indicators were tested via WB. The results revealed that treatment of rat pulmonary artery endothelial cells with si-NC and exposure to hypoxic conditions for 24 h obviously increased the protein expression levels of collagen I and III (representing extracellular matrix deposition), and this change was reversed by eIF3a knockdown by si-eIF3a intervention (Fig. 7A–C). Moreover, western blot results indicated that the expression of α-SMA and vimentin, which are mesenchymal and myofibroblasts markers, was clearly increased in pulmonary arterial endothelial cells exposed to hypoxia; however, the knockdown of eIF3a reversed the hypoxia-induced increase in the expression of α-SMA and vimentin (Fig. 7D–I). In addition, EndMT-related proteins (Snail, Slug and Twist1) were markedly increased in pulmonary arterial endothelial cells exposed to hypoxia, but in rat pulmonary artery endothelial cells, the upregulation of proteins related to EndMT induced by hypoxia was reversed by eIF3a knockdown (Fig. 7H–J). In agreement with the in vivo results, these findings suggested that the absence of eIF3a contributed to the attenuation of EndMT and alleviated the deposition of the extracellular matrix in a hypoxic environment.

### Alleviation of EndMT by eIF3a knockdown is associated with the TGFβ1/SMAD pathway

**Fig. 8:**
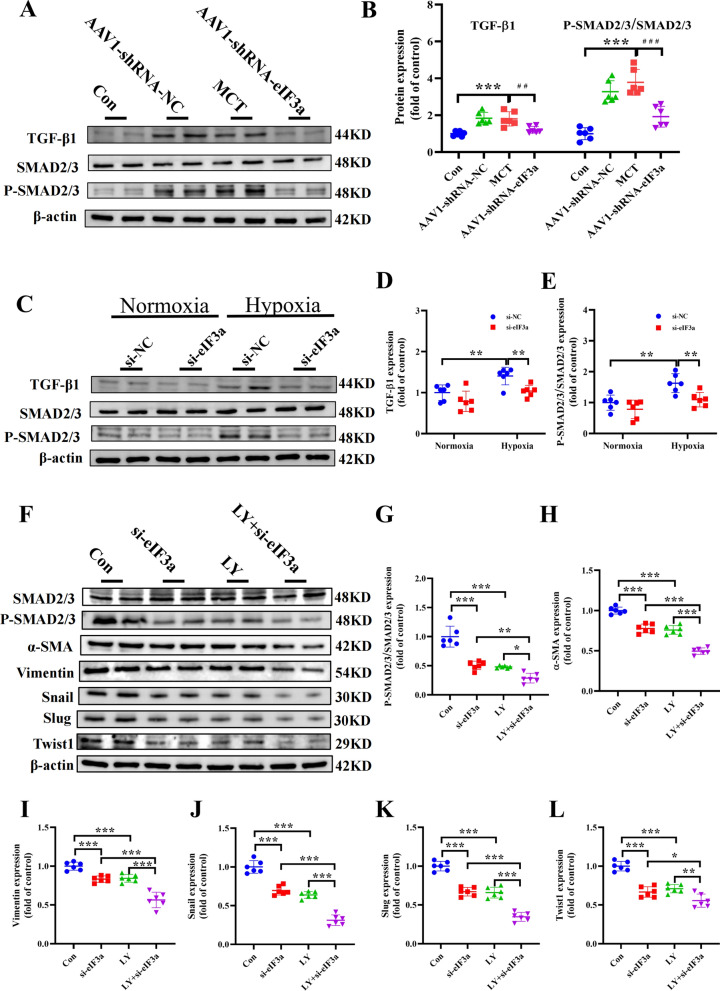
The potential role of the TGFβ1/SMAD pathway in the knockout of eIF3a to relieve EndMT. **A**, **B** In vitro experiment, western blot and the statistical results of TGFβ1 and p-SMAD2/3 proteins. **C**–**E** in vivo experiment, western blot and the statistical results of TGFβ1 and p-SMAD2/3 proteins. **F**–**L** in vivo experiment, western blot and the statistical results of TGFβ1 and p-SMAD2/3 proteins. Data are represented as the mean ± SD, n = 6–10, **P < 0.01, ***P < 0.001 vs. Control group, ^##^*P* < 0.01, ^###^*P* < 0.001 vs. MCT group

To elucidate the underlying mechanism by which eIF3a knockdown improves EndMT, we selected the TGFβ1/SMAD pathway, the most well-known signal involved in the regulation of EndMT, as the downstream factor. In the in vivo experiments, we observed that the protein expression level of TGFβ1 was significantly increased in the MCT group; moreover, as SMAD2/3 is an important protein regulated by TGFβ1, its phosphorylation level was significantly increased in the MCT group, and high levels of p-SMAD2/3 support this view. Notably, high expression of TGFβ1 and phosphorylation of SMAD2/3 in the rat pulmonary hypertension model were reversed by eIF3a knockdown, and the expression of these proteins was also significantly greater in the AAV1-shRNA-NC group than in the control group (Fig. 8A, B). Similarly, in vitro experiments revealed that cells cultured under hypoxia for 24 h presented increased levels of TGFβ1 expression and that the overexpression of TGFβ1 led to increased levels of p-SMAD2/3 compared with those in the normoxia group; however, this change was reversed in si-eIF3a-treated cells cultured under hypoxia for 24 h. In other words, EndMT and deposition of the extracellular matrix factors mentioned above increased along with the overexpression of eIF3a and the subsequent increase in p-SMAD2/3 (Fig. 8C–E). Furthermore, LY2109761 as a potent TGF-β type I/II receptor kinase inhibitor that suppresses Smad2/3 phosphorylation [39]. As shown in Fig. 8F–L, inhibition of TGF-β receptor kinase activity by LY2109761 markedly attenuated hypoxia-induced hyperphosphorylation of Smad2/3 and reversed the enhanced expression levels of mesenchymal markers α-SMA and vimentin, along with key epithelial-mesenchymal transition (EMT) regulators including Snail, Slug, and Twist1 proteins. And parallel effects were observed in eIF3a-knockdown models. PAECs treated with si-eIF3a exhibited substantially reduced Smad2/3 phosphorylation and diminished EndMT progression. Furthermore, combined treatment with LY2109761 and si-eIF3a synergistically amplified these effects, yielding even more pronounced suppression of Smad2/3 activation and EndMT marker expression compared to individual treatments. In short, the TGFβ1 and p-SMAD2/3 levels are positively related to the degree of eIF3a variation. These data revealed that the TGFβ1/SMAD pathway participated in the EndMT regulated by eIF3a to some extent.

## Discussion

**Fig. 9:**
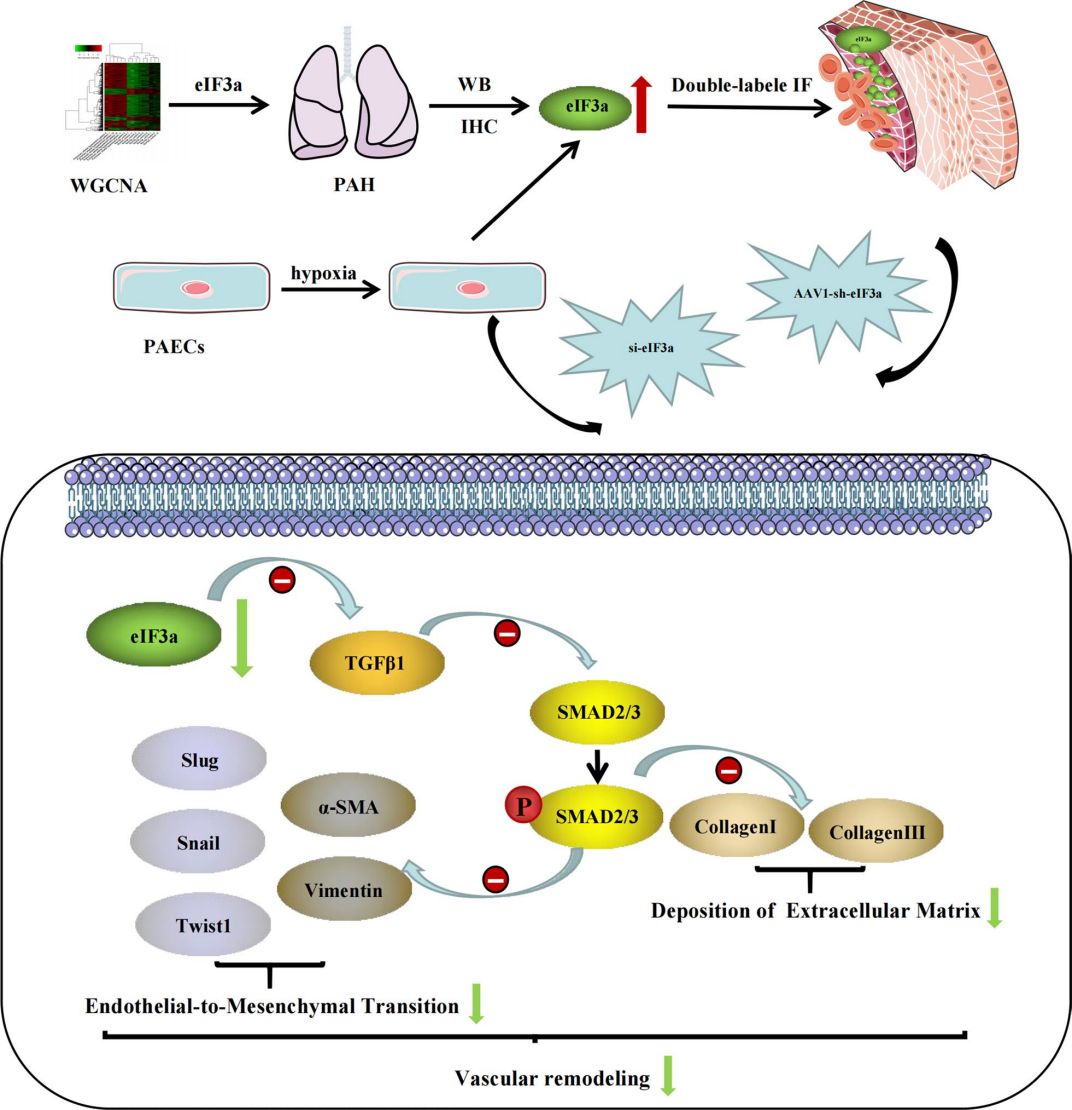
Schematic figure of the current study. The expression of eIF3a was significantly increased in MCT-induced pulmonary hypertension rat, double-labeled immunofluorescence showed eIF3a was mostly co-localized with CD31, indicated that the development of MCT-induced PAH was related to the regulation of pulmonary artery endothelial cell function. After knockdown of eIF3a expression by AAV1-shRNA-eIF3a, the expression of TGFβ1 was significantly down-regulated, and SMAD2/3 as a downstream molecule of TGFβ1 regulation, its phosphorylation level was significantly reduced, thus improving extracellular matrix deposition and EndMT in lung tissue of PAH rats, and improving vascular remodeling in pulmonary arteries of MCT-induced PAH rats. Moreover, in rat pulmonary artery endothelial cells incubated in a hypoxic environment, following the knockdown of eIF3a expression by si-eIF3a, we observed results consistent with the in vitro experiments

Our research revealed that eIF3a silencing suppresses the development of MCT-induced PAH by regulating EndMT in PAECs. The possible mechanism involves the TGFβ1/SMAD pathway, which is an important associated signal. This concept (Fig. 9) was based on the following evidence: (1) GEO data analysis suggested the differential expression of eIF3a in IPAH patients and normal controls. (2) Silencing eIF3a prevented MCT-induced PAH development in rats. (3) si-eIF3a inhibited EndMT in PAECs under hypoxic conditions, and (4) the activity of TGFβ1 and its downstream molecule SMAD was regulated by eIF3a in PAECs.

Pulmonary arterial hypertension (PAH) is a progressive disease of various origins. In PAH, the pulmonary vasculature is dynamically obstructed by vasoconstriction, structurally obstructed by adverse vascular remodeling, and pathologically noncompliant as a result of vascular fibrosis and stiffening [40–42]. Pulmonary vascular remodeling, which is caused mainly by changes in the intima, media and adventitial membrane, is the key structural alteration in pulmonary hypertension [5]. The vascular endothelium is the inner-most structure that coats the interior walls of arteries, capillaries and veins, and endothelial cells (ECs) anchor to an 80-nm-thick basal lamina (BL). The microenvironment of ECs is very dynamic and highly susceptible to mechanical stresses such as shear stress and cyclic stretching associated with pulsatile blood flow, as well as the composition and mechanical properties of the extracellular matrix (ECM). In addition, when ECs experience mechanical and biological changes in these microenvironments, the EC phenotype and functional change pathway are usually activated [43–45], and this process can cause extensive transcriptional reprogramming of activated endothelial cells, resulting in a shift to the mesenchymal cell phenotype and functional responses, called the endothelial-to-mesenchymal transition (EndMT) [46]. Numerous studies have shown that EndMT plays an important role in pulmonary fibrosis and pulmonary hypertension.

Eukaryotic initiation Factor 3 (eIF3) is one of the most complex translation initiation factors in mammalian cells and is composed of a multisubunit complex (eIF3a to eIF3 m), which is involved in various possible forms of messenger RNA (mRNA) translation, controlling every step of protein synthesis from initiation to elongation, termination, and quality control in both positive and negative manners [11, 47, 48]. eIF3a is the largest subunit of eIF3, which is a key player in all steps of translation initiation. Some studies have shown that eIF3a can promote TGFβ1-induced epithelial‒mesenchymal transition in alveolar epithelial cells [14]. Knockdown of eIF3a gene expression reversed TGFβ3a-induced proliferation of fibroblasts, thereby inhibiting the progression of bleomycin-induced pulmonary fibrosis in rats [49], eIF3a plays a crucial role in hypoxia-induced RV remodeling by regulating the TGF-β1-induced proliferation and differentiation of cardiac fibroblasts, which is mediated via the eIF3a/p27 pathway [16]. All the above studies showed that eIF3a participates in the occurrence of pulmonary fibrosis and right ventricular remodeling by regulating the expression of TGFβ1. Combined with the above findings, we hypothesized that eIF3a could relieve pulmonary hypertension in MCT-induced pulmonary hypertension rats by inhibiting EndMT, a process achieved by regulating the TGFβ1/SMAD signaling axis.

To explore the role of eIF3a in the development of pulmonary hypertension in MCT-induced PAH rats, a rat model of pulmonary hypertension was generated via the intraperitoneal injection of MCT, and both WB and immunohistochemistry revealed that eIF3a expression in the lung tissue was significantly greater in MCT-induced pulmonary hypertension rats than in control rats. In addition, rats with eIF3a silencing were treated with MCT, and 4 weeks later, hemodynamic detection and H&E staining revealed that the absence of eIF3a inhibited the development of PAH. Studies have shown that PAH is characterized by remodeling of the extracellular matrix of pulmonary arteries with increased collagen deposition, cross-linkage of collagen, and breakdown of elastic laminae. Notably, we found that the high expression of collagen I and collagen III, the main components of the extracellular matrix in the lung tissue of pulmonary hypertension rats, was clearly reversed by the absence of eIF3a, indicating that the knockdown of eIF3a inhibited extracellular matrix deposition in the lung tissues of MCT-induced pulmonary hypertension rats. To further explore the effect of EndMT on the pathological mechanism of pulmonary hypertension, WB experiments were performed, which revealed that the expression of α-SMA, vimentin, (mesenchymal markers), Twist1, snail and slug (endothelial to mesenchymal transition markers) was significantly increased in the lung tissue of MCT-induced pulmonary hypertension rats, but the absence of eIF3a reversed these changes. In vitro, we observed significantly increased expression of eIF3a, collagen I, collagen III, α-SMA, vimentin, Twist1, snail, and slug in pulmonary arterial endothelial cells exposed to hypoxia, which was reversed by the absence of eIF3a. These results indicate that eIF3a knockdown can reverse the occurrence of extracellular matrix deposition and endothelial interstitial transformation in MCT-induced pulmonary hypertension rats, and consistent results were observed in pulmonary artery endothelial cells exposed to hypoxia.

To explore the underlying mechanism by which eIF3a knockdown suppresses endothelial-to-interstitial translation, we focused on the TGFβ1/SMAD signaling pathway. Numerous studies have shown that the underlying mechanism of EndMT is the activation of the transforming growth factor-β, bone morphogenetic protein, Wingless/Integrated, or Notch signaling pathways [50–52]. Most of the signaling networks that are commonly utilized during EMT are also responsible for EndMT. The principal mediators are the TGF-β superfamily of proteins, including the isoforms TGF-β1 and TGF-β2, as discussed in a recent review. These signaling pathways interact with transcription factors, such as Snail, Slug, zinc finger E-box binding homeobox 1 (ZEB1), ZEB2 and Twist1, which induce mesenchymal cell gene expression but suppress endothelial gene expression in endothelial cells [53–55]. TGF-β family members trigger biological processes by inducing the formation of cell surface receptor complexes with intrinsic serine/threonine kinase activity. Seven human type I receptors (ALKs 1–7) and five human TGF-β family type II receptors, i.e., activin type II A and B receptors (ActRIIA and ActRIIB), BMP type II receptor (BMPRII), TGF-β type II receptor (TβRII), and AMH type II receptor (AMHRII), have been identified. By using different receptor complexes, ligands of the TGF-β family induce the phosphorylation and activation of specific R-Smads. Upon type I receptor activation, TGF-βs (via TβRI/ALK5) and activins (via ALK4/7) induce the phosphorylation of Smad2 and Smad3, followed by the formation of heteromeric complexes at Smad4. These complexes can translocate into the nucleus, where they regulate specific gene transcriptional responses, and Smad2/3 induce the expression of profibrotic genes [54, 56–58]. Tianyu et al. demonstrated that eIF3a silencing effectively suppresses the expression of TGF-β receptor type I (TGF-β RI) and type II (TGF-β RII) in responsive keloid fibroblasts (KFs). This inhibition subsequently reduces the phosphorylation levels of Smad2 and Smad3 induced by TGF-β1 stimulation. Through this molecular mechanism, eIF3a silencing ameliorates the pathological processes of keloid formation by attenuating two key pathogenic features: excessive KF cell proliferation and aberrant overproduction of ECM components [59]. Similarly, our experiments revealed that TGF-β1 expression and Smad2/3 phosphorylation were significantly increased in the MCT-induced pulmonary hypertension rat model, and the same results were observed in pulmonary arterial endothelial cells exposed to hypoxia, whereas eIF3a knockdown inhibited TGF-β1 expression, thereby inhibiting the phosphorylation of Smad 2/3.

## Conclusion

In this study, we proposed that in vivo knockdown of eIF3a attenuated MCT-induced PAH and vascular remodeling, a process achieved by modulating the TGF-β1/SMAD pathway and thus inhibiting the endothelial-to-mesenchymal transition. Furthermore, this phenomenon was further validated in rat pulmonary artery endothelial cells incubated in a hypoxic environment; therefore, our study suggests a potential role for eIF3a in MCT-induced PAH. The primary limitation of this study lies in the exclusive use of rat pulmonary artery endothelial cells (PAECs) and MCT-induced PAH rat models to investigate the role of eIF3a knockdown in endothelial‒mesenchymal transition and extracellular matrix deposition during MCT-induced pulmonary arterial hypertension. While hypoxic culture conditions have been employed to simulate PAH-related cellular injury in PAECs, primary human PAECs or patient-derived PAECs from PAH individuals are generally considered the gold standard for investigating pulmonary arterial pathophysiology under both normal and pathological conditions. Nevertheless, rat models have been extensively validated in PAH research and continue to provide valuable insights into disease mechanisms through both in vivo and in vitro experimental approaches. Furthermore, while numerous PAH animal models exist, no single preclinical model can fully recapitulate the complex spectrum of human PAH manifestations. Future investigations incorporating primary human PAECs, patient-derived cells, and diverse animal models (including transgenic systems) will be essential to validate the therapeutic potential of eIF3a knockdown in PAH vascular remodeling. Such comprehensive approaches will enable more robust verification of our preliminary findings regarding the inhibitory effects of eIF3a suppression on endothelial–mesenchymal transition through the modulation of the TGF-β1/SMAD signaling axis.

## Supplementary Information

Supplementary Material 1: Figure S1. DEGs in dodger blue module.

Supplementary Material 2: Figure S2. DEGs in dodger green module.

Supplementary Material 3: Figure S3. GO enrichment terms for DEGs in dodger blue module.

Supplementary Material 4: Figure S4. Differential expressed genes analysis.Histogram of connectivity distribution, and the scale-free topologywhen β = 29.Bar plots of mean absolute values of Gene Significanceacross modules. Higher mean GS suggests more significant associations between the module and IPAH.The Module Membershipversus Gene Significanceplots for blue and green module showed that MM and GS are highly correlated.

Supplementary Material 5: Figure S5.Statistical analysis of differential eIF3a expression using microarray dataset GSE15197.Statistical analysis of differential eIF3a expression using microarray dataset GSE53408.

## Data Availability

The datasets in this research are available on request to the corresponding author. The datasets GSE113439, GSE15197, GSE53408 could be downloaded from the NCBI Gene Expression Omnibus (GEO) database (http://www.ncbi.nlm.nih.gov/geo/).
