# Supplementary material for: Knockdown of eIF3a alleviates pulmonary arterial hypertension by inhibiting endothelial-to-mesenchymal transition via TGFβ1/SMAD pathway
Source: J Transl Med. 2025 May 9;23:524. doi: 10.1186/s12967-025-06505-3 (PMC12065328; doi:10.1186/s12967-025-06505-3)
Supplement: Supplementary file 1 — Supplementary Material 1: Figure S1. DEGs in dodger blue module. [file 12967_2025_6505_MOESM1_ESM.pdf]

ID	gene	logFC	AveExpr	t	P.Value	adj.P.Val	
8059393	CUL3	1.07325056	9.423328	10.47549	8.22E-09	3.04E-06	10.56208
8088700	TMF1	1.52592556	8.01E+00	9.69E+00	2.59E-08	4.94E-06	9.447922
8089930	GOLGB1	1.57191389	8.830913	10.709072	5.93E-09	2.66E-06	10.87785
8082940	ARMC8	1.22219	9.010326	10.458767	8.42E-09	3.06E-06	10.53924
8055913	PRPF40A	1.33791778	8.18E+00	8.66E+00	1.28E-07	1.03E-05	7.878274
7936614	EIF3A	1.53077	1.00E+01	9.69E+00	2.56E-08	4.92E-06	9.457976
8050302	ROCK2	1.61843444	9.193036	10.805074	5.19E-09	2.66E-06	11.00587
8043861	EIF5B	1.45228333	8.60E+00	8.72E+00	1.15E-07	9.82E-06	7.983122
7993664	CCP110	1.45478722	7.171216	11.09456	3.50E-09	2.46E-06	11.38583
7965022	KRR1	1.20302	8.39E+00	9.84E+00	2.06E-08	4.65E-06	9.670085
7929719	R3HCC1L	1.13096944	7.60E+00	9.86E+00	2E-08	4.65E-06	9.699941
8091562	DHX36	1.24966444	8.10E+00	9.21E+00	5.31E-08	6.74E-06	8.744311
8078569	GOLGA4	1.42817167	8.52E+00	8.87E+00	9.06E-08	8.83E-06	8.218727
8044304	RGPD6,RGPD1,RGPD5,RGPD2,RGPD8,RANBP2,RGPD4,RGPD3	1.03726111	1.06E+01	7.71E+00	6.21E-07	2.51E-05	6.315926
8054676	RGPD6,RGPD1,RGPD5,RGPD2,RGPD8,RANBP2,RGPD4,RGPD3	1.10574333	1.07E+01	7.93E+00	4.23E-07	2.04E-05	6.696301
7925622	AHCTF1,AHCTF1P1	1.32490611	8.28E+00	8.81E+00	1E-07	9.32E-06	8.119324
8046848	ZC3H15	1.41905444	9.355834	10.991169	4.03E-09	2.58E-06	11.25116
8043687	ANKRD36	1.34973944	7.771655	10.884392	4.66E-09	2.58E-06	11.11088
8120783	MYO6	1.04440778	9.50E+00	8.11E+00	3.14E-07	1.71E-05	6.992238
8020468	RBBP8	1.13659389	8.154115	10.963036	4.18E-09	2.58E-06	11.21432
8094638	WDR19	1.08253111	7.97E+00	8.60E+00	1.41E-07	1.08E-05	7.784904
7938313	IPO7	1.23684333	9.86E+00	8.57E+00	1.46E-07	1.10E-05	7.750192
8162147	ZCCHC6	1.366135	8.49E+00	9.04E+00	6.93E-08	7.81E-06	8.482259
8020508	RIOK3	1.08035	9.13E+00	8.51E+00	1.62E-07	1.17E-05	7.6437
8006112	NSRP1	1.56827278	8.179698	12.334358	7.03E-10	1.48E-06	12.91543
8152041	RNF19A	1.26106	9.63E+00	9.26E+00	4.97E-08	6.48E-06	8.809246
7974066	PNN	1.14609889	8.56E+00	9.96E+00	1.73E-08	4.33E-06	9.840609
8097480	NAA15	1.49834278	8.26E+00	9.35E+00	4.3E-08	6.08E-06	8.950526
8054414	RGPD1,RGPD6,RGPD5,RGPD2,RGPD8,RANBP2,RGPD4,RGPD3	1.11913444	1.07E+01	7.68E+00	6.55E-07	2.60E-05	6.262506
7929201	BTAF1	1.21408889	8.97E+00	7.35E+00	1.16E-06	3.64E-05	5.691832
8044161	RGPD1,RGPD6,RGPD5,RGPD2,RGPD8,RANBP2,RGPD4,RGPD3	1.07740667	1.06E+01	7.59E+00	7.72E-07	2.87E-05	6.100045
7901765	HOOK1	1.34016722	6.547711	10.895611	4.59E-09	2.58E-06	11.12568
8044745	DDX18	1.24789	8.66E+00	8.00E+00	3.81E-07	1.93E-05	6.799391
8056426	LOC100506124,TTC21B	1.39703222	7.48E+00	9.12E+00	6.13E-08	7.27E-06	8.602446
7965064	OSBPL8	1.41015389	9.11E+00	9.07E+00	6.59E-08	7.57E-06	8.531326
7954752	DNM1L	1.381055	8.70E+00	9.19E+00	5.46E-08	6.85E-06	8.716198
7968333	USPL1	1.06773889	8.42E+00	1.01E+01	1.45E-08	3.99E-06	10.00955
7946703	COPB1	1.28780111	9.39E+00	9.35E+00	4.32E-08	6.08E-06	8.946005
8045455	UBXN4	1.10395556	9.37E+00	8.04E+00	3.51E-07	1.82E-05	6.881331
8052269	CCDC88A	1.44226722	7.96E+00	9.72E+00	2.45E-08	4.80E-06	9.50225
8038919	ZNF350	1.03166667	7.23021	10.560266	7.30E-09	2.84E-06	10.67739
8105681	ERBB2IP	1.02480444	9.899717	11.2293	2.92E-09	2.26E-06	11.55962
7976598	PAPOLA	1.06587778	10.35094	12.384432	6.61E-10	1.48E-06	12.97406
8068062	USP16	1.49558889	8.70441	11.930126	1.17E-09	1.69E-06	12.43345
8112182	MIER3	1.18859333	7.78E+00	9.64E+00	2.76E-08	5.07E-06	9.384171
8148208	FAM91A1	1.12274667	9.515969	10.232129	1.16E-08	3.61E-06	10.22652
8131975	TAX1BP1	1.29692722	9.05E+00	9.33E+00	4.41E-08	6.14E-06	8.926304
7957806	SCYL2	1.14857778	9.76E+00	9.18E+00	5.56E-08	6.90E-06	8.698067
7957186	TBC1D15	1.24589222	9.57E+00	8.92E+00	8.34E-08	8.65E-06	8.299995

8107578	SRFBP1	1.37862889	8.550949	10.305956	1.05E-08	3.47E-06	10.32904
7971967	DIS3	1.31869167	8.00E+00	7.70E+00	6.33E-07	2.54E-05	6.29765
8084146	FXR1	1.14741722	8.64E+00	8.25E+00	2.47E-07	1.49E-05	7.230734
8056220	AHCTF1,AHCTF1P1	1.28557222	8.55E+00	9.52E+00	3.34E-08	5.37E-06	9.198893
8113914	FNIP1	1.04954444	9.14E+00	8.26E+00	2.45E-07	1.49E-05	7.238597
7925978	FAM208B	1.40047222	8.80E+00	7.37E+00	1.13E-06	3.58E-05	5.721773
8166230	TXLNG	1.29703611	8.75E+00	9.41E+00	3.92E-08	5.92E-06	9.040316
8020903	GALNT1	1.55108944	8.232718	12.677816	4.61E-10	1.30E-06	13.3129
7996954	NFAT5	1.00687111	9.91E+00	8.00E+00	3.78E-07	1.92E-05	6.80751
7975068	ZBTB1	1.07385389	8.33E+00	8.56E+00	1.49E-07	1.12E-05	7.728362
8129773	BCLAF1	1.40249722	9.24E+00	7.67E+00	6.64E-07	2.61E-05	6.249732
7983763	MAPK6	1.69067056	7.88E+00	9.75E+00	2.36E-08	4.66E-06	9.536184
7974920	SYNE2	1.25195667	8.85E+00	8.79E+00	1.03E-07	9.34E-06	8.095303
8043324	RGPD6,RGPD1,RGPD5,RGPD2,<br>RGPD8,RANBP2,RGPD4,RGPD3	1.07438222	1.01E+01	6.71E+00	3.75E-06	7.48E-05	4.525331
8053622	RGPD1,RGPD6,RGPD5,RGPD2,<br>RGPD8,RANBP2,RGPD4,RGPD3	1.07438222	1.01E+01	6.71E+00	3.75E-06	7.48E-05	4.525331
8106602	ZFYVE16	1.01380944	8.33E+00	1.01E+01	1.42E-08	3.98E-06	10.03119
7989253	SLTM	1.18931778	8.782823	10.721296	5.83E-09	2.66E-06	10.89421
7964937	ZFC3H1	1.14796167	8.76E+00	7.63E+00	7.13E-07	2.73E-05	6.178399
8113881	RAPGEF6	1.09519111	8.27E+00	8.29E+00	2.33E-07	1.45E-05	7.287301
7962590	RPAP3	1.41219611	6.711574	11.194138	3.06E-09	2.26E-06	11.51446
8049016	PSMD1	1.31850722	9.02E+00	8.07E+00	3.39E-07	1.79E-05	6.917462
7965723	UHRF1BP1L	1.00174167	8.025283	10.154936	1.30E-08	3.82E-06	10.11865
7929168	TNKS2	1.00111111	9.25E+00	7.10E+00	1.84E-06	4.90E-05	5.233131
8013906	TWF1P1,TWF1	1.18486389	8.915393	11.378306	2.40E-09	2.23E-06	11.7496
8081069	ZNF654	1.62057278	7.509995	10.95239	4.24E-09	2.58E-06	11.20036
7935146	NOC3L	1.245765	6.96E+00	7.30E+00	1.29E-06	3.90E-05	5.589298
7908459	CFH	1.62891778	9.69E+00	8.16E+00	2.88E-07	1.61E-05	7.078407
8042270	UGP2	1.02177167	8.92E+00	7.35E+00	1.18E-06	3.68E-05	5.678177
8044263	RANBP2	1.17686444	8.867105	10.281899	1.08E-08	3.49E-06	10.2957
7930276	SLK	1.39750667	9.75E+00	6.93E+00	2.49E-06	5.84E-05	4.934356
7981335	HSP90AA1	1.83067444	1.06E+01	8.40E+00	1.93E-07	1.31E-05	7.474553
8131479	MIOS	1.10026556	8.414837	11.223166	2.94E-09	2.26E-06	11.55175
7961654	RECQL	1.05584722	7.53E+00	7.51E+00	8.84E-07	3.09E-05	5.965026
7989885	LOC646358,DNAJB14	1.28393444	7.792513	11.785135	1.41E-09	1.76E-06	12.25672
7983663	USP8	1.72812944	8.612424	15.463918	2.07E-11	5.96E-07	16.17391
7962441	TWF1P1,TWF1	1.05328444	9.430579	10.898895	4.57E-09	2.58E-06	11.13001
7906819	ATF6	1.00952	1.06E+01	7.01E+00	2.16E-06	5.39E-05	5.074038
7988605	COPS2	1.35234667	9.83E+00	8.85E+00	9.38E-08	9.05E-06	8.184849
8157534	CNTRL	1.07591444	7.32E+00	9.26E+00	4.95E-08	6.48E-06	8.812626
8046975	WDR75	1.24028389	8.475805	10.381179	9.40E-09	3.24E-06	10.43284
7939197	HIPK3	1.22978667	1.01E+01	7.45E+00	9.79E-07	3.30E-05	5.863528
8107208	FER	1.20284111	8.17E+00	9.16E+00	5.77E-08	7.02E-06	8.661821
8094719	N4BP2	1.034055	7.10E+00	7.64E+00	7.06E-07	2.72E-05	6.188978
7977674	SUPT16H	1.19180833	8.85E+00	8.21E+00	2.64E-07	1.55E-05	7.16526
8150757	RB1CC1	1.25195222	8.03E+00	7.45E+00	9.78E-07	3.30E-05	5.865079
7967420	SBNO1	1.09929444	8.14E+00	9.80E+00	2.17E-08	4.66E-06	9.620842
8155770	SMC5	1.29609222	9.10E+00	7.78E+00	5.55E-07	2.36E-05	6.427188
8047401	CFLAR	1.67193667	10.09087	13.749773	1.31E-10	6.30E-07	14.48657
7956795	TBK1	1.14711778	8.14E+00	8.53E+00	1.56E-07	1.15E-05	7.683094
7968746	WBP4	1.03326167	6.93E+00	9.22E+00	5.26E-08	6.74E-06	8.75365
8095230	SRP72	1.15589	9.94E+00	6.91E+00	2.58E-06	5.95E-05	4.900648
7939314	EHF	1.53085444	9.32E+00	8.84E+00	9.5E-08	9.08E-06	8.171839

7930470	SHOC2	1.07770667	9.49E+00	9.79E+00	2.21E-08	4.66E-06	9.601248
8121949	LAMA2	1.65550778	9.13E+00	7.14E+00	1.69E-06	4.64E-05	5.318001
7917604	ZNF644	1.06928667	8.95E+00	8.90E+00	8.62E-08	8.78E-06	8.267509
8108134	DDX46	1.03931278	8.513494	11.112166	3.42E-09	2.46E-06	11.40865
8079305	EXOSC7,CLEC3B	-1.0477144	9.92E+00	-7.97E+00	3.96E-07	1.99E-05	6.763126
8020068	ANKRD12	1.13000278	9.14E+00	8.07E+00	3.35E-07	1.77E-05	6.928948
7922598	ANGPTL1	1.57084889	8.31E+00	1.00E+01	1.54E-08	4.06E-06	9.955294
8065730	EIF2S2	1.01987778	9.429883	11.484572	2.08E-09	2.08E-06	11.88369
8089234	ZBTB11	1.05170167	8.38E+00	6.90E+00	2.66E-06	6.07E-05	4.86991
8122440	LTV1	1.33753778	7.710577	11.48215	2.09E-09	2.08E-06	11.88064
8097647	ABCE1	1.24621	7.86E+00	8.71E+00	1.17E-07	9.87E-06	7.964348
8100615	UBA6	1.01442111	8.57E+00	8.99E+00	7.47E-08	8.07E-06	8.408581
7940372	TMEM109	-1.0235944	1.16E+01	-9.77E+00	2.29E-08	4.66E-06	9.567595
7958846	PTPN11	1.01010444	9.61E+00	7.42E+00	1.03E-06	3.38E-05	5.815265
7927854	HNRNPH3	1.15073056	8.741987	12.620878	4.94E-10	1.30E-06	13.24776
8130211	SYNE1	1.29258333	8.80E+00	9.70E+00	2.52E-08	4.90E-06	9.474149
7968577	NBEA	1.20049944	7.23E+00	7.95E+00	4.14E-07	2.03E-05	6.718392
7957043	FRS2	1.07943111	8.85E+00	9.13E+00	6.01E-08	7.19E-06	8.6221
7951008	SNORA40,TAF1D,MIR1304,SNORA1,<br>SNORA18,SNORA8,SNORD5,SNORA32	1.02063278	9.16E+00	8.06E+00	3.44E-07	1.79E-05	6.90274
8101659	SPARCL1	1.45586667	1.15E+01	6.72E+00	3.7E-06	7.42E-05	4.538339
7950990	SLC36A4	1.00813167	7.58E+00	9.50E+00	3.42E-08	5.48E-06	9.174948
7938687	NUCB2	1.15337222	8.47E+00	8.24E+00	2.55E-07	1.52E-05	7.198944
8053801	ANKRD36,ANKRD36C,ANKRD36BP2,<br>ANKRD36B	1.33331667	9.83E+00	9.12E+00	6.08E-08	7.25E-06	8.610287
8120552	FAM135A	1.10680833	8.45E+00	6.78E+00	3.32E-06	6.87E-05	4.649085
8019885	SMCHD1	1.04677778	9.48E+00	6.22E+00	9.51E-06	1.35E-04	3.597242
8008969	TBX2	-1.00395	9.51E+00	-6.84E+00	2.95E-06	6.41E-05	4.764576
8059413	DOCK10	1.42790667	8.59E+00	8.02E+00	3.69E-07	1.88E-05	6.832979
7917433	ODF2L	1.370405	7.55E+00	9.85E+00	2.02E-08	4.65E-06	9.688841
8007348	RAMP2	-1.8689944	9.52E+00	-7.55E+00	8.15E-07	2.94E-05	6.045909
7947245	HSP90AA2	1.64663778	7.60E+00	7.77E+00	5.56E-07	2.36E-05	6.425471
8106280	HMGCR	1.36173667	8.92E+00	6.17E+00	1.05E-05	1.45E-04	3.498428
8099760	ARAP2,LOC101928667	1.06212667	8.57E+00	6.89E+00	2.7E-06	6.14E-05	4.853939
8105714	SREK1	1.14210833	8.36192	10.101735	1.40E-08	3.96E-06	10.04391
8040440	GEN1	1.421285	6.79E+00	8.82E+00	9.82E-08	9.22E-06	8.140053
7956989	MDM2	1.03662444	9.61E+00	8.38E+00	2.02E-07	1.34E-05	7.430627
8034390	ZNF799	1.12100056	7.19E+00	7.36E+00	1.16E-06	3.63E-05	5.697289
7943498	DYNC2H1	1.00270722	5.59E+00	8.62E+00	1.36E-07	1.07E-05	7.817474
8103951	ACSL1	1.55306333	1.04E+01	6.09E+00	1.22E-05	1.60E-04	3.348666
8138466	MACC1	1.47640611	8.34E+00	8.21E+00	2.65E-07	1.56E-05	7.161469
8127841	PGM3	1.54846333	8.24E+00	6.66E+00	4.12E-06	7.98E-05	4.43243
8080685	SLMAP	1.02364444	8.792377	10.436244	8.69E-09	3.06E-06	10.50842
8142452	TFEC	1.25139833	7.40E+00	9.69E+00	2.55E-08	4.92E-06	9.460795
8006123	CPD	1.07957111	1.01E+01	6.00E+00	1.48E-05	1.82E-04	3.15862
7908488	CFHR1	1.310775	5.8604	10.633542	6.59E-09	2.67E-06	10.77641
7999520	RSL1D1	1.19261333	9.701232	10.907237	4.51E-09	2.58E-06	11.141
7973918	LOC101927178,FAM177A1	1.05650667	7.66E+00	7.38E+00	1.11E-06	3.54E-05	5.742684
8083941	ECT2	1.03628167	6.80E+00	6.83E+00	3E-06	6.44E-05	4.749871
7939902	LOC646813	1.13189611	7.07E+00	7.97E+00	3.99E-07	1.99E-05	6.755904
8104449	CCT5	1.12257333	9.07E+00	7.80E+00	5.31E-07	2.31E-05	6.470919
8113286	RIOK2	1.10724333	7.61E+00	7.83E+00	5.1E-07	2.27E-05	6.511762
8026182	MRI1	-1.0042511	9.21E+00	-7.61E+00	7.42E-07	2.80E-05	6.139509
8045619	EPC2	1.06402778	8.69E+00	9.54E+00	3.23E-08	5.37E-06	9.229335

7960794	CD163	1.91639444	9.25E+00	7.76E+00	5.69E-07	2.39E-05	6.403583
8174717	UPF3B	1.01509722	6.70E+00	7.52E+00	8.71E-07	3.08E-05	5.979945
7952243	C1QTNF5,MFRP	-1.0121667	9.19E+00	-6.47E+00	5.89E-06	9.76E-05	4.075273
8084064	MTHFD2P7,MTHFD2	1.34259222	8.77E+00	6.36E+00	7.3E-06	1.12E-04	3.861279
7951545	EXPH5	1.04967389	8.37E+00	8.57E+00	1.47E-07	1.11E-05	7.739598
8116910	HIVEP1	1.09806944	9.07E+00	6.27E+00	8.62E-06	1.25E-04	3.695652
8104079	FAT1	1.00625722	9.13E+00	6.46E+00	6.06E-06	9.90E-05	4.046689
8127977	SYNCRIP	1.03012222	9.54E+00	9.82E+00	2.13E-08	4.66E-06	9.636182
8053797	ANKRD36C	1.123	7.93E+00	6.99E+00	2.25E-06	5.51E-05	5.037502
8161737	RF00019	1.50491056	7.400992	11.821333	1.34E-09	1.76E-06	12.30103
8000799	GDPD3	-1.4723506	8.39E+00	-7.57E+00	8E-07	2.91E-05	6.064531
8152863	RF00402	1.01960278	6.99E+00	7.17E+00	1.62E-06	4.52E-05	5.362918
8026007	ZNF791	1.13959167	8.73E+00	6.99E+00	2.23E-06	5.50E-05	5.042308
8135392	HBP1	1.02711389	9.11E+00	5.87E+00	1.88E-05	2.16E-04	2.917434
7990138	GRAMD2	-1.0633667	8.80E+00	-7.88E+00	4.65E-07	2.16E-05	6.602606
8110166	HIGD2A	-1.04735	1.09E+01	-7.94E+00	4.2E-07	2.04E-05	6.703739
8138728	HOXA4	-1.3199844	7.82E+00	-6.32E+00	7.8E-06	1.17E-04	3.79529
7956670	MIR6125,USP15	1.13562778	8.95E+00	8.56E+00	1.5E-07	1.12E-05	7.720808
8058552	IDH1	1.30147833	9.56E+00	8.31E+00	2.27E-07	1.44E-05	7.314316
8042830	MTHFD2	1.56425111	9.15E+00	6.33E+00	7.73E-06	1.17E-04	3.804375
7962274	KIF21A	1.23007111	6.78E+00	6.84E+00	2.95E-06	6.41E-05	4.763808
8008982	TBX4	-1.1262294	9.87E+00	-8.25E+00	2.5E-07	1.51E-05	7.216928
8092224	RF00425	-1.1510717	6.607021	-10.66596	6.30E-09	2.66E-06	10.82003
7956878	IRAK3	1.39202	1.00E+01	5.96E+00	1.6E-05	1.93E-04	3.078215
8007701	EFTUD2,HIGD1B	-1.5817611	9.88E+00	-8.93E+00	8.22E-08	8.61E-06	8.31455
8122807	AKAP12	1.15239778	8.79E+00	6.63E+00	4.37E-06	8.28E-05	4.373429
7919642	HIST2H2AB	-1.0542244	9.52E+00	-6.22E+00	9.54E-06	1.35E-04	3.593979
8106516	JMY	1.01844111	8.25E+00	7.16E+00	1.63E-06	4.54E-05	5.356062
8135601	MET	1.376835	9.36E+00	7.76E+00	5.72E-07	2.39E-05	6.397916
8131067	GPR146	-1.6059672	8.93E+00	-9.26E+00	4.94E-08	6.48E-06	8.813805
8020149	NAPG	1.08074667	8.57E+00	5.95E+00	1.63E-05	1.95E-04	3.060946
8107613	SNX2	1.18631667	9.92E+00	8.68E+00	1.23E-07	1.02E-05	7.914091
8093906	WFS1	-1.0649133	9.90E+00	-9.16E+00	5.73E-08	7.00E-06	8.669688
7922889	IVNS1ABP	1.19711167	8.99E+00	6.43E+00	6.33E-06	1.02E-04	4.004211
8166876	DDX3X	1.25559556	1.02E+01	6.18E+00	1.02E-05	1.43E-04	3.523347
7968650	SERTM1	-1.0572889	9.08E+00	-8.12E+00	3.09E-07	1.69E-05	7.006725
8027760	FXYD1	-1.2031589	9.37E+00	-8.18E+00	2.8E-07	1.59E-05	7.107092
8038949	ZNF841	1.18003222	7.71E+00	6.46E+00	6.05E-06	9.90E-05	4.048239
7949615	B3GNT1	-1.0373872	7.75E+00	-5.97E+00	1.56E-05	1.89E-04	3.105765
8087925	TNNC1	-1.3370306	9.08067	-11.04357	3.75E-09	2.57E-06	11.31956
8046895	FAM171B	1.12102944	7.98E+00	8.88E+00	8.99E-08	8.83E-06	8.226341
7965964	SLC41A2	1.1986	8.48E+00	6.98E+00	2.28E-06	5.56E-05	5.020338
7965359	ATP2B1	1.04025167	8.89E+00	6.04E+00	1.35E-05	1.71E-04	3.250458
7924526	TP53BP2	1.01364444	9.42E+00	5.37E+00	5.13E-05	4.44E-04	1.914125
8172056	RPGR	1.29258778	7.83E+00	6.37E+00	7.12E-06	1.10E-04	3.885958
8075924	MFNG	-1.0808478	8.86E+00	-5.96E+00	1.59E-05	1.92E-04	3.084286
8143772	RARRES2	-1.3917339	9.881662	-11.67219	1.63E-09	1.83E-06	12.11761
8031646	ZNF667-AS1	-1.1905861	9.47E+00	-8.59E+00	1.43E-07	1.09E-05	7.767347
8101304	RASGEF1B	1.21345056	9.46E+00	5.87E+00	1.89E-05	2.17E-04	2.909682
8174474	ACSL4	1.23771222	9.07E+00	5.56E+00	3.5E-05	3.33E-04	2.296014
7898609	PLA2G5	-1.0004889	8.80E+00	-5.90E+00	1.8E-05	2.09E-04	2.961063
8057056	TTN	2.45350222	8.44E+00	7.92E+00	4.32E-07	2.06E-05	6.67608
7903777	GSTM5	-1.0898261	9.82E+00	-5.99E+00	1.5E-05	1.84E-04	3.142972
8097903	TLR2	1.13611944	8.72E+00	6.43E+00	6.4E-06	1.03E-04	3.991992

7926410	MRC1	1.33233222	1.04E+01	6.70E+00	3.85E-06	7.60E-05	4.500327
7926451	MRC1	1.33233222	1.04E+01	6.70E+00	3.85E-06	7.60E-05	4.500327
8157524	TLR4	1.10458444	9.93E+00	6.93E+00	2.51E-06	5.86E-05	4.926327
8047300	AOX1	1.54964167	8.41E+00	5.77E+00	2.3E-05	2.50E-04	2.71323
7952004	SCN4B	-1.3512683	8.26E+00	-8.89E+00	8.73E-08	8.79E-06	8.255745
8103722	HSP90AA6P	1.90984056	6.54E+00	8.02E+00	3.66E-07	1.87E-05	6.840614
8000687	NPIPB4,LOC613037,SMG1P7,SMG1P5, LOC102725125,NPIPB5,SLC7A5P1, SMG1, BOLA2,NPIPA5,SMG1P2,SMG1P3, SMG1P1,LOC101060386, LOC101060604,LOC102723773	1.0381	1.11E+01	5.44E+00	4.45E-05	3.98E-04	2.055769
8089596	WDR52	1.24616444	7.74E+00	7.91E+00	4.41E-07	2.09E-05	6.656035
7954382	PYROXD1	1.28664889	7.76E+00	8.72E+00	1.15E-07	9.82E-06	7.980253
8117402	HIST1H4E	-1.0359211	9.89E+00	-5.24E+00	6.76E-05	5.44E-04	1.639476
8041206	LBH	-1.38412	1.06E+01	-6.98E+00	2.3E-06	5.59E-05	5.013713
8131967	HOXA-AS2	-1.014925	6.33E+00	-5.27E+00	6.31E-05	5.17E-04	1.707175
8093258	IQCG	1.01505667	7.58E+00	6.79E+00	3.25E-06	6.82E-05	4.667604
7927732	ARID5B	1.00201222	1.01E+01	5.03E+00	0.000104	7.47E-04	1.207972
7922408	SNORD78	1.39034833	7.51E+00	6.71E+00	3.78E-06	7.52E-05	4.519082
8111941	HMGCS1	1.27677	8.80E+00	5.05E+00	9.96E-05	7.24E-04	1.252633
8123739	NRN1	-1.1168967	9.84E+00	-5.48E+00	4.18E-05	3.79E-04	2.118588
8041570	TMEM178A	-1.1446211	9.19E+00	-6.77E+00	3.34E-06	6.90E-05	4.641262
8009334	CACNG4	-1.0875683	9.23E+00	-6.65E+00	4.2E-06	8.08E-05	4.412715
8014316	CCL5	-1.2755783	1.03E+01	-6.43E+00	6.34E-06	1.02E-04	4.00152
8096917	NONHSAT097842	1.088395	5.36E+00	5.80E+00	2.16E-05	2.39E-04	2.779375
8070557	ZBTB21	1.13926333	8.85E+00	5.81E+00	2.12E-05	2.36E-04	2.79613
7920875	SCARNA4	-2.337835	8.68E+00	-9.30E+00	4.63E-08	6.30E-06	8.877525
8106743	VCAN	1.4266	9.91E+00	4.99E+00	0.000114	8.00E-04	1.117347
7932407	ST8SIA6	-1.0965222	8.27E+00	-7.29E+00	1.31E-06	3.92E-05	5.575246
7922418	SNORD44,SNORD79,SNORD77,GAS5,SNORD74, SNORD81,SNORD80,SNORD47,SNORD76	-1.3230267	10.88392	-10.71849	5.85E-09	2.66E-06	10.89045
8135661	CFTR	1.14350611	7.61E+00	6.13E+00	1.13E-05	1.52E-04	3.424167
8044049	IL18RAP	1.32374556	7.59E+00	4.79E+00	0.000173	1.10E-03	0.701072
8062490	SNORA60	-1.1864789	1.08E+01	-8.47E+00	1.74E-07	1.22E-05	7.576179
8133770	CCDC146	1.08815556	7.95E+00	7.87E+00	4.7E-07	2.17E-05	6.593059
8117372	HIST1H2AC	-1.1149489	8.94E+00	-7.40E+00	1.08E-06	3.47E-05	5.769646
8100378	RPL21P44	1.34434	7.02E+00	5.84E+00	2.01E-05	2.26E-04	2.850092
8097335	HSPA4L	1.04642833	6.70E+00	4.30E+00	0.000493	2.47E-03	-0.33679
8134051	C7orf63	1.03284389	6.92E+00	5.26E+00	6.47E-05	5.27E-04	1.6823
8013567	LYRM9	-1.0270422	5.93E+00	-8.03E+00	3.6E-07	1.85E-05	6.855809
8166500	ZFX	1.06072389	8.92E+00	7.44E+00	9.98E-07	3.33E-05	5.844707
7914807	NA	-2.1040522	5.62E+00	-7.67E+00	6.64E-07	2.61E-05	6.249518
8138718	HOXA2	-1.1081733	7.62E+00	-5.08E+00	9.5E-05	7.01E-04	1.300023
7943413	BIRC3	1.583545	8.82E+00	4.73E+00	0.000195	1.21E-03	0.582809
8013341	MFAP4	-1.0223056	1.19E+01	-7.10E+00	1.83E-06	4.86E-05	5.241675
8151871	CCNE2	1.08359889	6.17E+00	5.62E+00	3.15E-05	3.08E-04	2.401869
7969091	RNY3P2	1.02065444	6.90E+00	4.75E+00	0.00019	1.18E-03	0.611762
7934434	MYOZ1	-1.9484067	7.67E+00	-6.38E+00	7.01E-06	1.09E-04	3.902216
8144786	SLC7A2	1.21261444	9.54E+00	7.13E+00	1.74E-06	4.69E-05	5.291728
7903049	CCDC18	1.04627111	5.29E+00	6.21E+00	9.7E-06	1.37E-04	3.577247
8138735	HOXA5	-1.0728456	8.55E+00	-5.07E+00	9.69E-05	7.12E-04	1.280038
7903389	NA	1.04561722	8.14E+00	7.45E+00	9.75E-07	3.30E-05	5.867771
8149324	FAM167A	-1.0544411	8.11E+00	-6.96E+00	2.35E-06	5.66E-05	4.993631
8059279	EPHA4	1.03390778	8.38E+00	5.51E+00	3.9E-05	3.61E-04	2.187204

8154233	CD274	1.13261111	8.15E+00	4.12E+00	0.000726	3.35E-03	-0.71884
7901495	NA	-1.0404367	4.71E+00	-6.67E+00	4.05E-06	7.87E-05	4.4502
7951038	SNORA40,TAF1D,MIR1304,SNORA1, SNORA18,SNORA8,SNORD5,SNORA32	1.11011222	8.22E+00	4.37E+00	0.000423	2.20E-03	-0.18447
7999384	PP4135	1.05848	1.03E+01	5.81E+00	2.11E-05	2.36E-04	2.799458
8124531	HIST1H3I	-1.5668611	9.26E+00	-6.62E+00	4.47E-06	8.38E-05	4.351229
8146957	PI15	1.96691167	6.19E+00	5.86E+00	1.94E-05	2.20E-04	2.883398
8151559	SLC10A5	1.29745667	6.06E+00	7.82E+00	5.19E-07	2.29E-05	6.494754
8065427	GGTLC1	-1.0873922	8.67E+00	-6.64E+00	4.32E-06	8.23E-05	4.383984
8043236	GNLY	-1.1390467	8.30E+00	-3.88E+00	0.001214	5.05E-03	-1.22608
7919151	RNVU1-10	-1.0696172	1.02E+01	-7.93E+00	4.26E-07	2.04E-05	6.689361
7919392	RNVU1-10	-1.0696172	1.02E+01	-7.93E+00	4.26E-07	2.04E-05	6.689361
8150889	SDR16C5	1.09650611	9.39E+00	5.44E+00	4.49E-05	4.00E-04	2.047549
7989146	MNS1	1.08616333	6.11E+00	5.38E+00	5.03E-05	4.38E-04	1.933501
7942135	ANO1	-1.014305	9.21E+00	-7.17E+00	1.62E-06	4.52E-05	5.365686
8038809	NKG7	-1.282215	9.26E+00	-3.89E+00	0.001194	4.98E-03	-1.20928
8171418	PIGA	1.18058222	8.49E+00	5.36E+00	5.26E-05	4.53E-04	1.889514
8078225	RNU4-85P	1.31823889	6.21E+00	5.41E+00	4.73E-05	4.17E-04	1.995575
8064894	LRRN4	-1.1988778	1.01E+01	-7.13E+00	1.74E-06	4.70E-05	5.289149
8125919	FKBP5	1.60556333	1.07E+01	4.04E+00	0.000856	3.82E-03	-0.88157
8056611	LRP2	1.21873556	7.93E+00	4.39E+00	0.000406	2.13E-03	-0.14425
8116952	RNU1-11P	-1.137305	9.93E+00	-6.75E+00	3.51E-06	7.17E-05	4.590984
8035838	ZNF724P	1.06517611	5.65E+00	4.94E+00	0.000126	8.62E-04	1.019701
7982854	DLL4	-1.0028144	9.70E+00	-6.27E+00	8.65E-06	1.26E-04	3.691942
7970954	DCLK1	1.22493722	7.34E+00	5.33E+00	5.6E-05	4.74E-04	1.826087
7984540	KIF23	1.090865	6.55E+00	7.65E+00	6.88E-07	2.67E-05	6.214848
7934898	ANKRD22	1.46041722	6.46E+00	5.25E+00	6.65E-05	5.37E-04	1.656098
8142646	IQUB	1.24093389	5.25E+00	4.39E+00	0.000408	2.14E-03	-0.14914
8105267	ITGA2	1.29591444	9.44E+00	3.67E+00	0.001922	7.31E-03	-1.67643
8144569	RNU6-729P	1.00407111	7.07E+00	4.57E+00	0.000276	1.58E-03	0.238523
8151030	RN7SKP97	1.00315167	5.27E+00	4.52E+00	0.000306	1.71E-03	0.136491
7980152	LTBP2	-1.0048778	1.09E+01	-6.63E+00	4.36E-06	8.28E-05	4.37494
8122058	ARG1	1.12713889	4.99E+00	3.51E+00	0.002712	9.65E-03	-2.01294
8108627	VTRNA1-1	-1.4644644	9.09E+00	-6.62E+00	4.44E-06	8.36E-05	4.358255
7983630	FGF7	1.02524167	6.83E+00	3.60E+00	0.002228	8.22E-03	-1.82109
8043043	DNAH6	1.30312944	6.73E+00	4.19E+00	0.000617	2.94E-03	-0.55831
8138363	SOSTDC1	-1.6605028	7.87E+00	-4.13E+00	0.000711	3.30E-03	-0.69842
7910950	KMO	1.00072167	7.17E+00	4.00E+00	0.000943	4.12E-03	-0.97724
