# Supplementary material for: Knockdown of eIF3a alleviates pulmonary arterial hypertension by inhibiting endothelial-to-mesenchymal transition via TGFβ1/SMAD pathway
Source: J Transl Med. 2025 May 9;23:524. doi: 10.1186/s12967-025-06505-3 (PMC12065328; doi:10.1186/s12967-025-06505-3)
Supplement: Supplementary file 2 — Supplementary Material 2: Figure S2. DEGs in dodger green module. [file 12967_2025_6505_MOESM2_ESM.pdf]

ID	gene	logFC	AveExpr	t	P.Value	adj.P.Val	
7948606	TMEM258	-1.094083333	11.1730333	-7.516420797	8.72E-07	3.08E-05	5.979021765
8031097	NDUFA3	-1.166879444	10.1300593	-8.285075433	2.35E-07	1.45E-05	7.280671773
8161147	HINT2	-1.202036667	10.2269853	-6.838205617	2.96E-06	6.41E-05	4.760966851
7940996	PRDX5	-1.231647222	10.7833967	-9.623329671	2.84E-08	5.15E-06	9.35660636
8097056	SNHG8,SNORA24	-1.030277222	9.64460133	-6.270376441	8.66E-06	0.000125779	3.69078168
7934706	NA	1.024798889	8.605784	6.98767563	2.25E-06	5.52E-05	5.035064241
8045804	NA	-1.521332222	10.6274893	-7.044873973	2.03E-06	5.19E-05	5.139108726
8084213	SNORA63D	-1.265318889	7.83789133	-6.06814653	1.28E-05	0.000165705	3.29879651
8025584	SNORD105	-1.077813889	7.63048	-6.968495733	2.33E-06	5.63E-05	5.000070932
7947989	RF00019	1.072995	8.14822133	7.032951275	2.07E-06	5.26E-05	5.117459832
7905088	HIST2H2AC	-1.386650556	9.55628867	-7.920759839	4.33E-07	2.06E-05	6.674043053
8000706	CDIPT	-1.003816667	10.1560733	-6.156716827	1.08E-05	0.000147473	3.471159832
8075462	SELM	-1.081022778	10.6552353	-7.759500286	5.71E-07	2.39E-05	6.399618984
7971920	HNRNPA3P9,HNRNPA3P10,HNRNPA3, HNRNPA3P1,HNRNPA3P12,HNRNPA3P3, HNRNPA3P6,HNRNPA3P4,HNRNPA3P5， HNRNPA3P15	1.385188889	9.88195333	10.93090686	4.37E-09	2.58E-06	11.17214164
8012000	HSPC254	-1.033271667	8.47607133	-5.717091108	2.57E-05	0.000268485	2.605381054
7911337	MT-TA	-1.472638889	11.8946	-7.227123034	1.46E-06	4.22E-05	5.467497887
7973871	MT-TA	-1.472638889	11.8946	-7.227123034	1.46E-06	4.22E-05	5.467497887
8165696	MT-TA	-1.472638889	11.8946	-7.227123034	1.46E-06	4.22E-05	5.467497887
7999419	DEXI	-1.156280556	8.82550667	-7.443884505	9.91E-07	3.31E-05	5.851887934
8030364	SNORD33,RPL13A,SNORD35A, SNORD34,SNORD32A	-1.250433333	9.20693	-6.614559719	4.50E-06	8.39E-05	4.344895321
8083445	RF00019	1.375713889	9.61411	7.043060577	2.03E-06	5.19E-05	5.135817326
7981960	SNORD116-6	-1.038293333	8.88727267	-6.528193275	5.29E-06	9.12E-05	4.182319808
8156358	NA	-1.236193333	7.73763933	-6.461864029	6.00E-06	9.85E-05	4.056748362
8124406	HIST1H2BE,HIST1H2BF, HIST1H2BG,HIST1H2BI,HIST1H2BC	-1.3926	7.16258333	-5.914476186	1.73E-05	0.000203747	2.997247411
8013523	NA	-1.321542222	5.15305533	-7.591147207	7.65E-07	2.85E-05	6.109211523
8165709	MT-TP	-1.087566111	10.407318	-4.284371847	0.000507	0.002523186	-0.36414643
8045287	NA	-1.061167778	9.08865067	-5.286395646	6.15E-05	0.000507759	1.733651711
8124397	HIST1H1C	-1.083409444	8.679604	-5.481706365	4.13E-05	0.000375262	2.131680933
8017098	NONHSAT054977	-1.017017222	7.239212	-5.086591067	9.29E-05	0.000690216	1.322150274
8127656	RF00012	1.275374444	6.39570533	5.200326087	7.34E-05	0.000579917	1.556902341
8073680	NA	-1.181362778	8.326636	-3.942759748	0.001059	0.004519859	-1.09130053
7899502	RNU11	-1.002438889	9.00780667	-3.536352242	0.002553	0.00918601	-1.95394225
7915612	PTCH2,RNU5E-6P	-1.11689	5.92746733	-4.580764741	0.000269	0.001546327	0.263931974
7913655	ID3	-1.043547778	9.45477867	-3.773778175	0.001527	0.006085547	-1.45082012
7906443	FCER1A	-1.273746111	7.580236	-4.59124532	0.000263	0.001519835	0.286055592
