# Supplementary material for: Knockdown of eIF3a alleviates pulmonary arterial hypertension by inhibiting endothelial-to-mesenchymal transition via TGFβ1/SMAD pathway
Source: J Transl Med. 2025 May 9;23:524. doi: 10.1186/s12967-025-06505-3 (PMC12065328; doi:10.1186/s12967-025-06505-3)
Supplement: Supplementary file 3 — Supplementary Material 3: Figure S3. GO enrichment terms for DEGs in dodger blue module. [file 12967_2025_6505_MOESM3_ESM.pdf]

Table1: GO analysis of the DEGs (top 30)

Terms	Count	ratio	PValue	Genes
GO:0000042 protein targeting to Golgi	8	2.5806	6.77E-09	RGPD6, RGPD5, RGPD8, RGPD2, RGPD1, RGPD4, RGPD3, GOLGA4
GO:0009615 response to virus	8	2.5806	9.48E-04	IRAK3, CCT5, DDX3X, TBK1, DHX36, CCL5, IVNS1ABP, DCLK1
GO:0032728 positive regulation of interferon-beta production	4	1.2903	0.00632	DDX3X, TBK1, TLR2, TLR4
GO:0035721 intraciliary retrograde transport	3	0.9677	0.00828	WDR19, TTC21B, DYNC2H1
GO:2001275 positive regulation of glucose import in response to insulin stimulus	3	0.9677	0.01397	RARRES2, OSBPL8, PTPN11
GO:0098609 cell-cell adhesion	10	3.2258	0.01506	RSL1D1, TWF1, ZC3H15, MYO6, USP8, DDX3X, SLK, SNX2, IDH1, HIST1H3I
GO:0006886 intracellular protein transport	9	2.9032	0.01912	RAMP2, TBC1D15, RPGR, MYO6, NAPG, IPO7, COPB1, SNX2, EXPH5
GO:0018108 peptidyl-tyrosine phosphorylation	7	2.2581	0.02155	EPHA4, HSP90AA1, FGF7, MET, CNTRL, FER, TTN
GO:0010501 RNA secondary structure unwinding	4	1.2903	0.02404	DDX18, DDX46, DDX3X, DHX36
GO:0030900 forebrain development	4	1.2903	0.027	DYNC2H1, LRP2, FRS2, DCLK1
GO:0005654 nucleoplasm	63	20.323	4.69E-05	KIF23, COPS2, TNNC1, TAF1D, SHOC2, SYNCRIP, WBP4, PNN, et al
GO:0005829 cytosol	67	21.613	6.89E-04	KIF23, TNNC1, TBK1, EIF5B, NBEA, FER, TTN, GSTM5, EIF3A,et al
GO:0016020 membrane	46	14.839	0.00357	MFNG, GALNT1, FKBP5, NAA15, DNAJB14, SYNCRIP, EIF3A,et al
GO:0005794 Golgi apparatus	23	7.4194	0.00377	GALNT1, CCDC88A, DNM1L, FGF7, USP8, MYO6, RPGR, TLR2,et al
GO:0005640 nuclear outer membrane	4	1.2903	0.00413	TMEM109, SYNE1, SYNE2, NUCB2
GO:0005737 cytoplasm	91	29.355	0.00596	COPS2, TBK1, ANO1, NAA15, EIF5B, TLR2, SHOC2, EIF3A,et al
GO:0005635 nuclear envelope	8	2.5806	0.0063	ATF6, SYNE1, SYNE2, NUCB2, MNS1, RANBP2, UBXN4, TNKS2
GO:0016607 nuclear speck	9	2.9032	0.00653	BCLAF1, DDX46, DDX3X, EFTUD2, NOC3L, NSRP1, WBP4, PNN, PRPF40A
GO:0070062 extracellular exosome	54	17.419	0.00815	RARRES2, LTBP2, ANO1, ATP2B1, CUL3, CFHR1, HIST2H2AB,et al
GO:0005741 mitochondrial outer membrane	7	2.2581	0.01667	EPHA4, ARG1, ACSL1, DNM1L, DDX3X, KMO, ACSL4
GO:0044822 poly(A) RNA binding	36	11.613	3.04E-06	BCLAF1, NOC3L, EIF5B, NAA15, SYNCRIP, NSRP1, PNN, EIF3A, et al
GO:0005524 ATP binding	40	12.903	4.37E-05	KIF23, BTAF1, TBK1, UBA6, FER, TTN, RIOK2, DNAH6, ATP2B1, et al
GO:0005515 protein binding	141	45.484	0.00204	RARRES2, LTBP2, WFS1, TBK1, TNNC1, HMGCR, EIF5B, EIF3A, et al
GO:0004713 protein tyrosine kinase activity	8	2.5806	0.00215	EPHA4, TWF1, HSP90AA1, FGF7, MET, CNTRL, FER, TTN
GO:0005085 guanyl-nucleotide exchange factor activity	7	2.2581	0.00523	DIS3, RPGR, RAPGEF6, RASGEF1B, DOCK10, ECT2, FNIP1
GO:0046982 protein heterodimerization activity	14	4.5161	0.01042	HIST1H2AC, SNX2, TLR2, ITGA2, TXLNG, FXR1, ATF6, CUL3, HIST2H2AB, IRAK3, MAPK6, HIST1H4E, ZBTB1, HIST1H3I
GO:0016887 ATPase activity	8	2.5806	0.01202	KIF23, ABCE1, HSP90AA1, DDX3X, DYNC2H1, CFTR, KIF21A, DNAH6
GO:0098641 cadherin binding involved in cell-cell adhesion	10	3.2258	0.01686	RSL1D1, TWF1, ZC3H15, MYO6, USP8, DDX3X, SLK, SNX2, IDH1, HIST1H3I
GO:0004674 protein serine/threonine kinase activity	11	3.5484	0.03131	IRAK3, SLK, MAPK6, RIOK3, ROCK2, TBK1, HIPK3, SMG1, TTN, RIOK2, DCLK1
GO:0003723 RNA binding	14	4.5161	0.03458	EXOSC7, SYNCRIP, FXR1, DIS3, RSL1D1, PAPOLA, EIF3A, HNRNPH3, DDX18, DDX3X, EIF2S2, HBP1, RANBP2, PRPF40A

List Total	Pop Hits	Pop Total	foldEnrichment	Bonferroni	Benjamini	FDR	Category
238	20	16792	28.22184874	9.37E-06	9.37E-06	1.11E-05	GO_BP
238	110	16792	5.131245225	0.7307982	0.481153	1.548	GO_BP
238	27	16792	10.45253657	0.9998461	0.946411	9.91093	GO_BP
238	10	16792	21.16638655	0.99999	0.943769	12.7919	GO_BP
238	13	16792	16.28183581	1	0.979612	20.6576	GO_BP
238	271	16792	2.603491581	1	0.969777	22.0884	GO_BP
238	236	16792	2.690642359	1	0.977988	27.2088	GO_BP
238	153	16792	3.227989235	1	0.976931	30.1267	GO_BP
238	44	16792	6.414056532	1	0.976299	32.9951	GO_BP
238	46	16792	6.135184509	1	0.977361	36.2592	GO_BP
250	2784	18224	1.649586207	0.0138459	0.013846	0.06262	GO_CC
250	3315	18224	1.473312821	0.1850868	0.097275	0.91527	GO_CC
250	2200	18224	1.524189091	0.6546003	0.298371	4.66346	GO_CC
250	863	18224	1.942767092	0.6742695	0.244535	4.91424	GO_CC
250	24	18224	12.14933333	0.7070733	0.217739	5.36659	GO_CC
250	5222	18224	1.27030563	0.8304386	0.256032	7.66253	GO_CC
250	159	18224	3.66772327	0.8469214	0.23518	8.08576	GO_CC
250	201	18224	3.264	0.8570142	0.215827	8.36697	GO_CC
250	2811	18224	1.400350053	0.9119412	0.236599	10.3409	GO_CC
250	149	18224	3.424644295	0.993222	0.393109	20.0969	GO_CC
228	1129	16881	2.360868957	0.0011993	0.001199	0.00423	GO_MF
228	1495	16881	1.980989262	0.0171276	0.008601	0.06087	GO_MF
228	8785	16881	1.188339874	0.5535351	0.235703	2.80228	GO_MF
228	133	16881	4.453502176	0.5732925	0.191774	2.95722	GO_MF
228	118	16881	4.392172168	0.8738644	0.339052	7.0376	GO_MF
228	465	16881	2.229145444	0.9840487	0.498275	13.5721	GO_MF
228	183	16881	3.236698303	0.991572	0.494553	15.4939	GO_MF
228	290	16881	2.553085299	0.9987879	0.568039	21.0769	GO_MF
228	376	16881	2.166048432	0.9999965	0.75248	35.7845	GO_MF
228	547	16881	1.894977389	0.9999991	0.750923	38.7331	GO_MF
