# Supplementary figures and images for: Knockdown of eIF3a alleviates pulmonary arterial hypertension by inhibiting endothelial-to-mesenchymal transition via TGFβ1/SMAD pathway
Source: J Transl Med. 2025 May 9;23:524. doi: 10.1186/s12967-025-06505-3 (PMC12065328; doi:10.1186/s12967-025-06505-3)

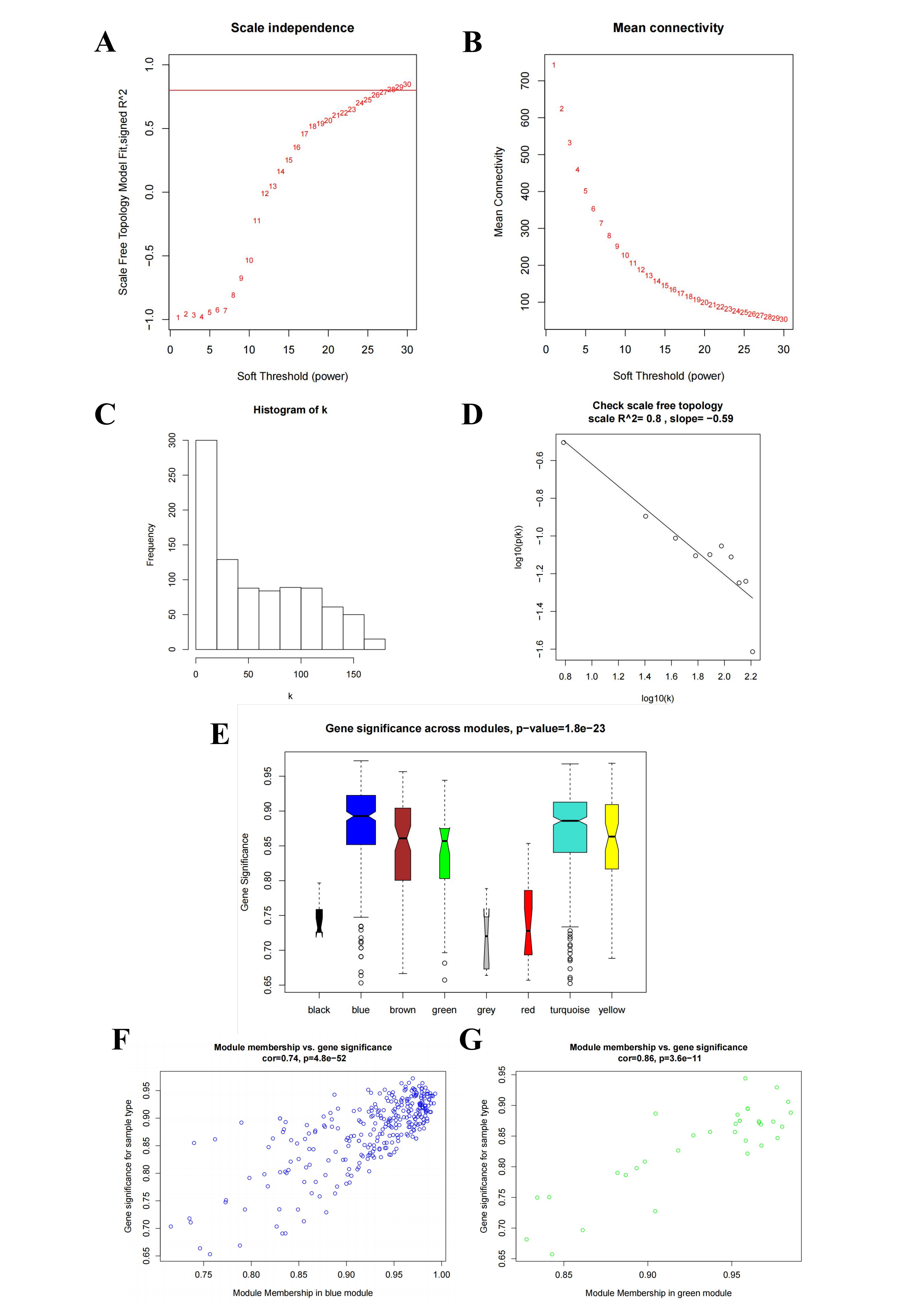

Supplement: Supplementary file 4 — Supplementary Material 4: Figure S4. Differential expressed genes analysis.Histogram of connectivity distribution, and the scale-free topologywhen β = 29.Bar plots of mean absolute values of Gene Significanceacross modules. Higher mean GS suggests more significant associations between the module and IPAH.The Module Membershipversus Gene Significanceplots for blue and green module showed that MM and GS are highly correlated. [file 12967_2025_6505_MOESM4_ESM.tif]

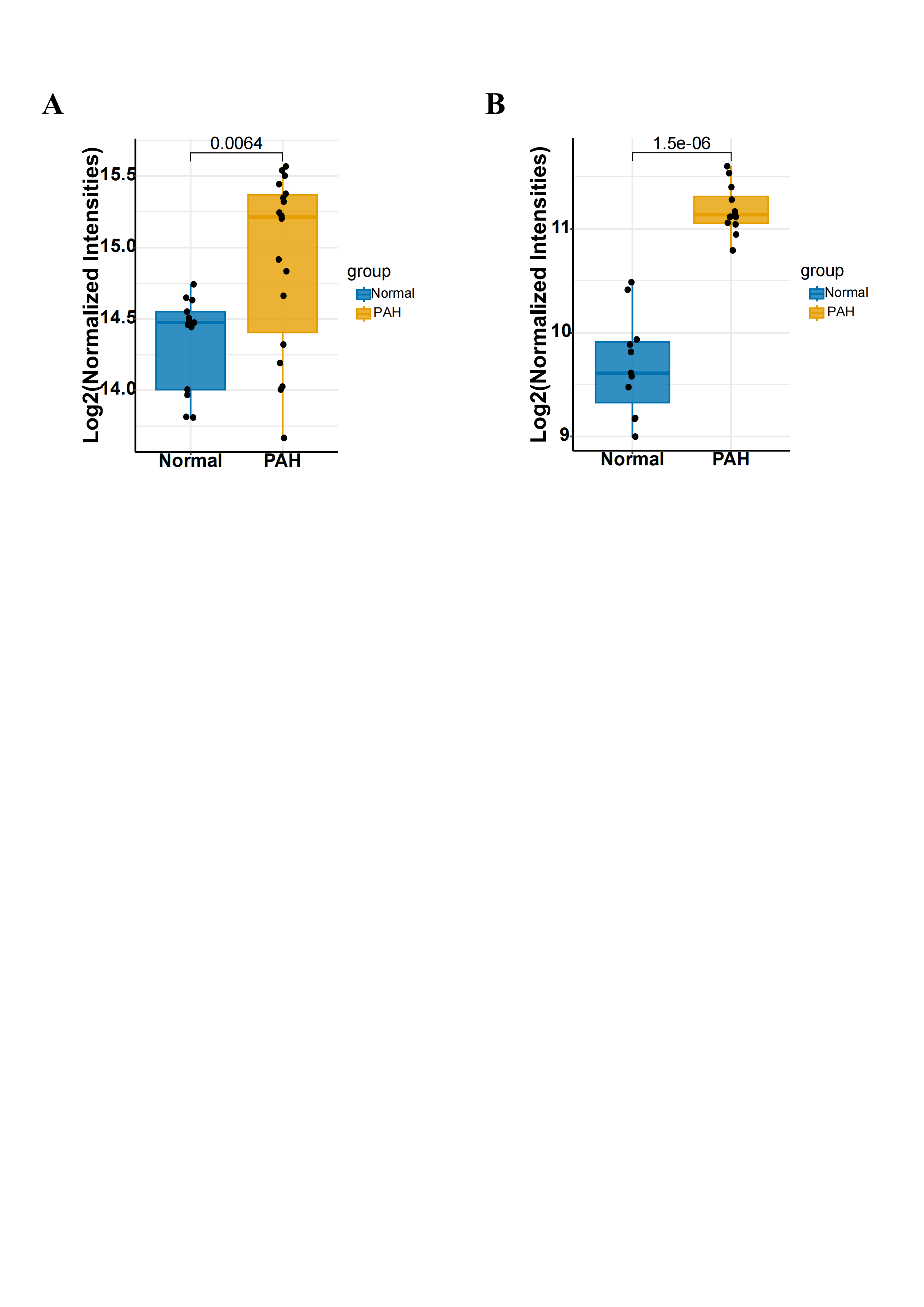

Supplement: Supplementary file 5 — Supplementary Material 5: Figure S5.Statistical analysis of differential eIF3a expression using microarray dataset GSE15197.Statistical analysis of differential eIF3a expression using microarray dataset GSE53408. [file 12967_2025_6505_MOESM5_ESM.tif]
